# Fast seasonal growth promotes regional stability by strengthening local temporal stability in Inner Mongolian grasslands

**DOI:** 10.3389/fpls.2026.1902582

**Published:** 2026-07-23

**Authors:** Zexu Nie, Yujie Yuan, Hushun Zhang, Fang Shi, Han Zhang, Chao Wang

**Affiliations:** 1 Key Laboratory of Ecological Impacts of Hydraulic-Projects and Restoration of Aquatic Ecosystem of Ministry of Water Resources, Institute of Hydroecology, Ministry of Water Resources and Chinese Academy of Sciences, Wuhan, China; 2 School of Urban Design, Wuhan University, Wuhan, China; 3 National Permanent Scientific Research Base for Warm Temperate Zone Forestry of Jiulong Mountain in Beijing, Experimental Center of Forestry in North China, Chinese Academy of Forestry, Beijing, China

**Keywords:** climate change, grassland, multidimensional stability, regional stability, seasonal growth characteristics

## Abstract

**Introduction:**

Grassland productivity stability underpins the sustained functioning of arid and semi-arid ecosystems yet identifying how regional stability emerges across local communities remains challenging because stability is multidimensional, scale- dependent, and is shaped by seasonal growth processes.

**Methods:**

Here, we use satellite-derived gross primary productivity (GPP) data from 2000 to 2018 to investigate how local stability, spatial synchrony, drought resistance, recovery, and fast-growing phase characteristics jointly regulate regional stability across meadow, typical, and desert steppes in Inner Mongolia and across three observational grains (0.1°, 0.5°, and 1.0°).

**Results:**

We found that regional stability was governed primarily by local stability rather than spatial synchrony, suggesting that the stabilizing role of spatial insurance was limited under broad-scale climatic constraints. At the local scale, temporal stability was more closely aligned with resistance than with recovery, while resistance and recovery showed a persistent trade-off. Mechanistically, growth rate during the fast-growing phase (FGR) linked environmental variation to stability by reflecting how efficiently vegetation converted favorable hydrothermal conditions into mean GPP. Higher FGR strengthened local temporal stability mainly through higher mean GPP, while its relationships with resistance and recovery depended on productivity variability and post-drought resource conditions. Observational grain further modified the expression of stability and the detectability of relationships among stability dimensions.

**Discussion:**

These findings identify fast seasonal growth as a key pathway through which favorable hydrothermal windows are translated into local stability, with consequences for regional stability across observational grains. They also suggest that grassland monitoring and management should consider the spatial grain at which stability is assessed.

## 1 Introduction

Grassland ecosystem stability is critical for sustaining ecosystem functions under climatic variability, including forage production, biodiversity conservation, and carbon sequestration (Bai et al., 2004; Yu et al., 2025). This issue is especially important at regional scales, where ecosystem responses have more direct implications for management and conservation (Cheng and Li, 2024). Classical theory suggests that regional stability can be enhanced when asynchronous dynamics among local communities compensate for one another in space, making spatial insurance a major pathway for maintaining ecosystem functioning at broad scales (Wang and Loreau, 2014; Wilcox et al., 2017). However, in grasslands, climatic fluctuations, particularly variation in water availability, may strengthen synchrony among local communities and thereby constrain the stabilizing role of spatial insurance (Wang et al., 2020b). As a result, how regional stability is maintained, and whether the contribution of spatial insurance changes across climatic gradients, remain insufficiently understood.

To understand how regional stability emerges, it is first necessary to clarify how local stability is transmitted across scales. At the local scale, ecosystem stability cannot be treated as a single property, but it is increasingly recognized as multidimensional, encompassing not only temporal stability but also resistance to and recovery from disturbances (Isbell et al., 2015; Hillebrand et al., 2018; Kéfi et al., 2019; Chen et al., 2023). Temporal stability reflects the consistency of ecosystem functioning over time, resistance describes the ability to maintain functioning during disturbances, and recovery indicates the capacity to regain functioning after stress (Donohue et al., 2013). Although often interrelated, these dimensions may couple, decouple, or trade off depending on ecological strategies and environmental conditions (Polazzo and Rico, 2021; Patrick et al., 2022). For instance, communities dominated by acquisitive species may exhibit rapid recovery but low resistance, while conservative communities often display strong resistance but slower recovery (Yan et al., 2025). Such variation reveals the complex ecological processes regulating ecosystem persistence under stress. However, despite growing recognition of multidimensional stability, we still lack a systematic understanding of how environmental drivers regulate distinct stability dimensions, particularly across expansive spatial scales and various climatic regimes.

A further step is to identify the vegetation processes that regulate productivity dynamics. In grasslands, growing attention has been directed toward seasonal growth characteristics, particularly phenology and growth rate, which represent plant adaptations to optimize growth and reproduction under variable climatic conditions (Wang et al., 2020a; Liu et al., 2025). By regulating the timing, intensity, and duration of biomass accumulation, these growth characteristics influence not only the magnitude of productivity but also its interannual variability (Wang et al., 2020a; Hallmark et al., 2024; Mentges et al., 2024; Yang et al., 2025). These regulatory effects suggest that vegetation growth characteristics may shape multidimensional stability through their effects on productivity dynamics. Theoretically, a higher growth rate is widely hypothesized to increase ecosystem productivity and facilitate faster recovery after disturbance, at the potential cost of increasing the sensitivity of productivity to environmental fluctuations, thereby reducing resistance (de Bello et al., 2021; Tao et al., 2024). In contrast, slower and more conservative growth may buffer disturbance but constrain regrowth potential (Wright et al., 2010; Reich, 2014). Beyond altering local trade-offs, seasonal growth characteristics may scale up to shape regional stability by modulating spatial synchrony among constituent communities. By altering the timing and pace of vegetation growth, phenology and growth rate can change how local communities respond to climatic variation, potentially increasing asynchronous dynamics and thereby contributing to regional stability (Simmonds et al., 2020; Hallmark et al., 2024). Together, these dynamics suggest that seasonal growth characteristics may shape ecosystem stability not only by regulating productivity dynamics and trade-offs among local stability dimensions, but also by influencing synchrony among local communities. However, empirical evidence remains limited regarding whether these mechanisms operate in a consistent or context-dependent manner across climatic gradients. Clarifying these pathways is therefore essential for understanding how grassland stability emerges from local processes to regional stability under climatic variability.

**Figure 1:**
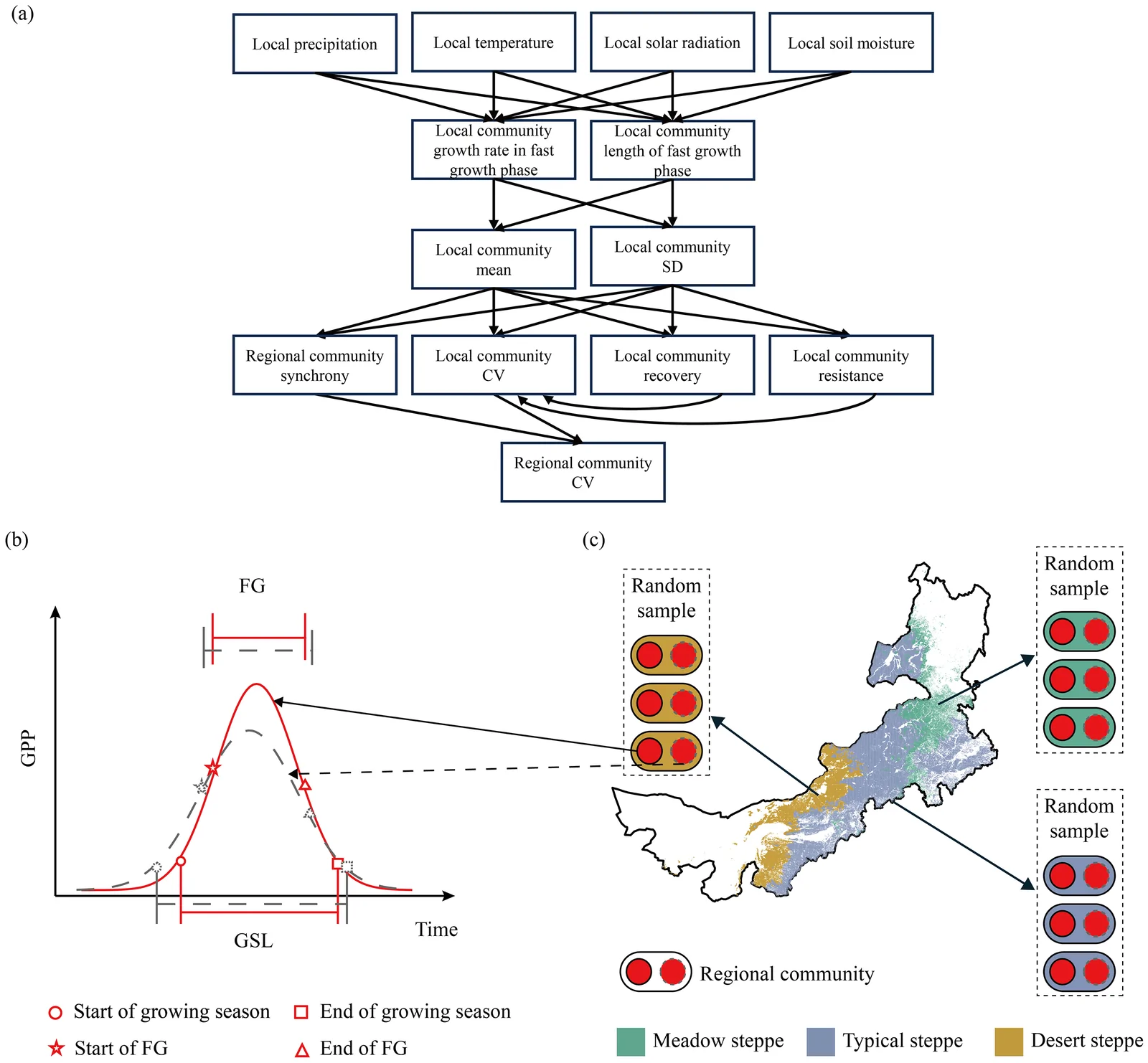
Integrated framework linking local seasonal growth to regional stability. The mechanistic framework **(a)** illustrates how local seasonal environmental factors shape local community growth dynamics and influence regional stability. The schematic GPP curve **(b)** shows the identification of the growing season and the fast-growing phase and highlights differences in growth patterns of local communities. The illustration **(c)** demonstrates the aggregation of local communities into regional communities, based on random sampling across three steppe types: meadow, typical, and desert steppes. GPP, gross primary productivity.

To address these gaps, we developed a hierarchical framework integrating environmental factors, seasonal growth characteristics, multidimensional stability at the local scale, and regional stability (**Figure 1a**). Using satellite-derived gross primary productivity (GPP) data, we quantified growth rate (FGR) and the length (FGL) of the fast-growing phase to examine how these seasonal growth characteristics were associated with productivity dynamics, local stability, and regional stability. We further evaluated whether these relationships varied across three observational grains (0.1°, 0.5°, and 1.0°), and three steppe types (meadow, typical, and desert steppes). Specifically, we address the following research questions: (1) Is regional stability governed more strongly by local stability or by spatial synchrony among communities? (2) How are temporal stability, resistance, and recovery related to one another across climatic contexts and observational grains? (3) Do seasonal growth characteristics provide a mechanistic pathway linking environmental variation to ecosystem stability across scales? By integrating regional stability, multidimensional local stability, and seasonal growth processes within a single framework, this study advances our understanding of how grassland ecosystems maintain stability under climatic variability.

## 2 Materials and methods

### 2.1 Study area

The Inner Mongolian grassland, located in northern China, constitutes a major component of the Eurasian steppe. It has a temperate continental monsoon climate, characterized by cold, dry winters and warm, humid summers (You et al., 2023). From 2000 to 2018, the region had an average annual precipitation of 328.1 mm and a mean annual temperature of 4.3 °C (Nie et al., 2025). Our study area encompassed three distinct steppe types: meadow, typical, and desert (**Figure 1**). The desert steppe represents the warmest and most arid environment, whereas the meadow steppe represents the coolest and most humid zone (**Supplementary Figure 1**).

Grassland extent was identified using the MODIS Terra/Aqua Combined Land Cover Type product (MCD12C1, Collection 6; 0.05° resolution). Based on the International Geosphere-Biosphere Programme (IGBP) classification scheme, pixels consistently classified as grassland during 2001–2018 were extracted as the grassland mask (spatial data for 2000 were excluded due to satellite unavailability). Steppe types were determined using a 30-m map from the Institute of Grassland Survey and Planning of Inner Mongolia, which was produced from Landsat 5/7 TM images acquired during the peak growing season in 2009 and supported by field surveys. The final study area was obtained by overlaying the MODIS-derived grassland mask with the steppe-type map, thereby identifying meadow, typical, and desert steppes.

### 2.2 Extraction and calculation of gross primary productivity data

The study utilized an 8-day, 0.05° vegetation photosynthesis model GPP dataset covering the period from 2000 to 2018. Each calendar year included 46 data entries, calculated as 8-day averages, except for the 46th entry, which was calculated as a 5-day or 6-day average depending on whether it was a leap year. Based on enhanced light-use efficiency theory, the dataset integrates satellite data from MODIS and climate data from the National Centers for Environmental Prediction (Reanalysis II). By incorporating type-specific parameters for C3 and C4 plants, it achieves higher accuracy in estimating vegetation photosynthesis and minimizes errors from low-quality or missing data through a gap-filling and smoothing algorithm (Zhang et al., 2017).

Annual productivity at the pixel scale was calculated by summing the accumulated GPP for all periods within a year. For the first 45 periods, the raw data values were multiplied by eight, while for the 46th period, the values were multiplied by five (or six in a leap year).

### 2.3 Estimation of parameters to describe the fast-growing phase

To identify the fast-growing phase, defined as the period during which GPP remains relatively high and stable within the growing season, we first reconstructed the annual GPP curve at each pixel using a two-step smoothing approach. First, the original 8-day GPP time series was denoised and interpolated using a Savitzky-Golay filter, which helps reduce short-term fluctuations while preserving phenological metrics (Yang et al., 2019). Second, we fitted the smoothed GPP data with a generalized additive model (GAM) from the ‘*mgcv*’ package in R to produce an annual growth curve. In this model, GPP was the response variable, and the day of year (DOY) was the predictor. Thin plate regression splines were employed for smoothing, with the base dimension parameter *k* set to its default (Wood, 2017).

From the fitted GPP curve, we identified the start of the fast-growing phase as the day when the first derivative of GPP with respect to time reached its maximum, and the end of this phase was similarly defined as the subsequent day when the first derivative reached its minimum. We then quantified the growth rate of fast-growing phase (FGR) as the mean fitted daily GPP during this phase, which represents the average carbon uptake rate:


$$FGR_{i,y}=\frac{\sum_{t\in T_{i,y}}\hat{GPP_{i,y}}\left(t\right)}{FGL_{i,y}}$$

where *T_i, y_* is the set of days within the fast-growing phase, $\hat{GPP_{i,y}}\left(t\right)$ is the fitted daily GPP on day *t*. *FGL_i, y_* was calculated as the number of days between these two inflection points (Wang et al., 2020a).

### 2.4 Definition of climate extremes and multidimensional stability

To identify climate extremes at the pixel level, we analyzed annual precipitation data spanning 1978–2018. For each pixel, annual precipitation anomalies were standardized using the pixel-specific long-term mean and standard deviation (SD) of annual precipitation over 1978–2018. A year was classified as dry if the standardized precipitation anomaly was lower than −1.28, and as wet if it was higher than 1.28, corresponding approximately to the lower and upper 10% tails of a standardized normal distribution. Years between −1.28 and 1.28 were classified as normal. Thus, drought and wet years were identified using pixel-specific standardized anomalies rather than a uniform absolute precipitation threshold across the study area. The 1978–2018 precipitation record was used only to define the local climatic background for identifying anomalously dry and wet years. Resistance and recovery were calculated only for drought events that occurred during the GPP observation period from 2000 to 2018. Because GPP in wet years consistently exceeded normal-year levels, wet years did not represent functional declines relative to normal conditions. Resistance and recovery were therefore evaluated only for drought events. In cases of consecutive dry years, resistance was calculated for each dry year, whereas recovery was quantified only in the first non-dry year following the drought.

Temporal stability was assessed using the coefficient of variation (CV) of annual GPP over 2000–2018, calculated as the standard deviation divided by the mean; lower CV indicates greater temporal stability. Resistance and recovery were calculated as:


$$Resistance=\frac{{\bar{Y}}_{n}}{\left|Y_{d-}Y_{n}\right|}$$

$$Recovery=\frac{\left|Y_{d}-Y_{n}\right|}{\left|Y_{d+1}-Y_{n}\right|}$$

Here, *Y_n_*, *Y_d_*, and *Y_d_* _+_ _1_ represent GPP during normal years within 2000–2018 (average over all normal years), during a dry year, and one year after a dry year, respectively (Chen et al., 2023). To reduce heteroscedasticity and improve comparability among metrics, both resistance and recovery were log-transformed and then averaged across all identified drought events. Higher values of resistance and recovery indicate greater stability under drought stress.

### 2.5 Construction of regional communities

To upscale local dynamics to the regional level, we constructed regional communities by pairing local pixels using a simulated landscape approach (van der Plas et al., 2016; Hautier et al., 2020; Wang et al., 2022). In this framework, each pixel represented a local community, and two local communities were randomly combined without replacement to form one simulated regional community (**Figure 1c**). Pairwise combinations were used because they maximized the number of regional communities that could be generated within each steppe type while avoiding overlap among local communities within a given iteration, thereby providing sufficient sample size for subsequent analyses. For each steppe type, all available pixels were included in the resampling pool, and each iteration generated the maximum possible number of non-overlapping pairs. For example, in meadow steppe, 11 pixels yielded 5 regional communities per iteration, with one pixel left unpaired. To reduce sensitivity to any single random configuration, the pairing procedure was repeated 500 times, resulting in 500 replicate datasets for each steppe type.

### 2.6 Upscaling local temporal stability to regional scales

To quantify temporal stability at the regional scale, we applied a theoretical framework that links regional stability to local stability and spatial synchrony among local communities (Wang and Loreau, 2014; Wang et al., 2019). This relationship is defined as:


$$CV_{R}=CV_{L}\times \varphi_{R}$$

where *CV_R_* and *CV_L_* represent temporal stability at the regional and local scales, respectively, with lower CV indicating higher temporal stability. Synchrony (*φ_R_*) captures the similarity of interannual fluctuations between paired local communities. Higher *φ_R_* indicates stronger synchrony, and it is calculated as:


$$\varphi_{R}=\frac{\sqrt{\sum_{ij}v_{ij}^{L}}}{\sum_{i}\sqrt{v_{ii}^{L}}}$$

where $v_{ij}^{L}$ is the temporal covariance between local communities *i* and *j*, and $v_{ii}^{L}$ represents the temporal variance of local community *i*.

### 2.7 Environmental factors

We obtained daily gridded climate data, including precipitation, air temperature, and solar radiation, from the China Meteorological Forcing Dataset (CMFD), which has a spatial resolution of 0.1° and is derived from ground-based observations and multiple gridded data sources (He et al., 2020). Daily soil moisture data were obtained from the Soil Moisture of China by *in situ* data, version 1.0 (SMCI1.0), which provides 1-km-resolution estimates for 10 vertical layers (0–100 cm) based on a random forest model trained on observations from 1, 789 stations (Li et al., 2022). We focused on the soil moisture in the upper 0–30 cm because more than 80% of plant roots in the Inner Mongolian grasslands are distributed within this depth (Ma et al., 2008). Soil moisture for 0–30 cm was calculated as the mean of the 0–10 cm, 10–20 cm, and 20–30 cm layers. To examine how environmental conditions influenced vegetation seasonal growth, we calculated the mean values of precipitation, air temperature, solar radiation, and soil moisture during the fast-growing phase for each pixel and year.

To test whether the observed ecological relationships were robust to variations in spatial scaling, all fast-growing phase metrics and environmental variables were harmonized and aggregated from their baseline resolutions into three coarser observational grains (0.1°, 0.5°, and 1.0°) using arithmetic averaging. These different resolutions do not represent spatial scale in the sense of ecological hierarchy (local vs. regional) but instead reflect different levels of spatial data grain size.

### 2.8 Statistical analysis

We used a multi-step analytical framework to assess how environmental drivers and vegetation growth characteristics influence ecosystem stability at both local and regional scales. At the local scale, linear regression models were first used to examine how environmental variables influenced two seasonal growth characteristics: growth rate and the length of the fast-growing phase. These characteristics were then included as predictors in subsequent regressions linking them to annual productivity metrics, specifically the mean and standard deviation of GPP. Finally, we evaluated how these productivity characteristics were associated with multidimensional stability. Relationships between temporal stability, resistance, and recovery were further examined using Pearson’s correlation coefficients.

At the regional scale, we investigated how local-scale metrics and regional community synchrony jointly influenced the regional CV. To capture hierarchical pathways linking environmental conditions, seasonal growth characteristics, local stability, and regional stability, we constructed a piecewise structural equation model (SEM) based on the conceptual framework (**Figure 1a**). Hypothesized causal pathways were fitted using linear models, and path coefficients were estimated with the ‘*piecewiseSEM*’ package (Lefcheck, 2016).

All models shared the same theoretical backbone linking regional CV, regional synchrony, local CV, resistance, recovery, local mean GPP, local GPP standard deviation, FGR, and FGL. Because the environmental controls of seasonal growth may differ among steppe types and observational grains, the environmental predictors of FGR and FGL were selected separately for each steppe type × grain-size combination. Candidate environmental models including precipitation, temperature, soil moisture, and solar radiation were compared using AIC (Akaike information criterion), and the model with the lowest AIC and clear ecological interpretability was retained.

To account for the stochastic nature of regional community construction, we adopted a resampling-based inference approach. In each iteration, regional communities were formed from non-overlapping pairs of local communities, and the final path model was fitted separately. This procedure was repeated 500 times for each steppe type × grain-size combination. For each final model, we summarized the AIC distribution across the 500 randomized model runs using the mean, 2.5th percentile, and 97.5th percentile (**Supplementary Table 1**). For each path, we calculated the mean standardized coefficient, median standardized coefficient, and the 2.5th and 97.5th percentiles of the coefficient distribution. Path robustness was evaluated using the proportion of coefficients with signs opposite to the mean coefficient (**Supplementary Tables 2**–**S10**). A path was considered robust when this opposite-sign proportion was< 0.05 and marginally robust when it was< 0.10 (Wang et al., 2022). We also summarized the R² values of each component linear model across the 500 runs to quantify the proportion of variance explained for each response variable (**Supplementary Tables 11**–**S13**).

To examine differences in vegetation growth characteristics among meadow, typical, and desert steppes, we performed a one-way analysis of variance (ANOVA). All analyses and figures were conducted using R version 4.3.3 (R Development Core Team, 2024).

## 3 Results

### 3.1 Patterns of multidimensional stability across steppe types and observational grains

**Figure 2:**
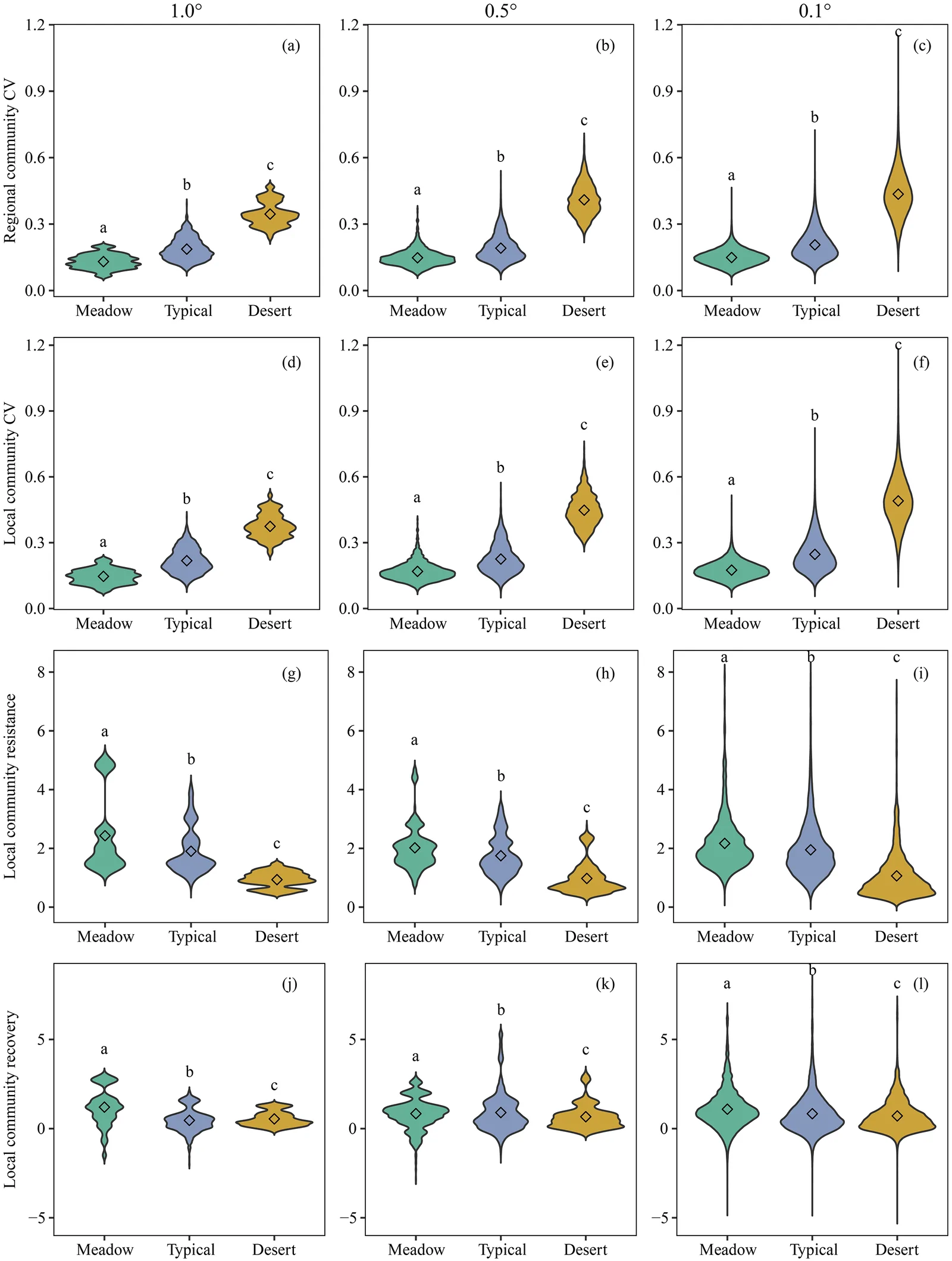
Comparisons of multidimensional stability among steppe types and observational grains. Panels **(a–c)** show regional community CV, **(d–f)** local community CV, **(g–i)** local community resistance, and **(j–l)** local community recovery. Different letters above the violin plots indicate significant differences among steppe types within the same observational grain (*p* < 0.05). CV: coefficient of variation.

We compared multidimensional stability across meadow, typical, and desert steppes at both regional and local scales under three observational grains (1.0°, 0.5°, and 0.1°) (**Figures 2a–c**). At the regional scale, meadow steppe consistently exhibited the lowest CV, indicating the highest temporal stability, whereas desert steppes showed the greatest variability. Typical steppes were intermediate across all grains. These patterns remained consistent, although finer grains revealed greater within-type variability.

At the local scale, CV displayed the same pattern among steppe types as at the regional scale (**Figures 2d–f**). Resistance likewise was highest in meadow steppes and lowest in desert steppes. By contrast, recovery exhibited a clear grain-dependent pattern: at 0.1°, recovery declined from meadow to typical to desert steppe; at 0.5°, typical steppe showed the highest recovery; and at 1.0°, meadow steppe ranked highest again, whereas desert steppes showed slightly higher recovery than typical steppes (**Figures 2j–l**).

### 3.2 Relationships among local stability dimensions across steppe types and observational grains

**Figure 3:**
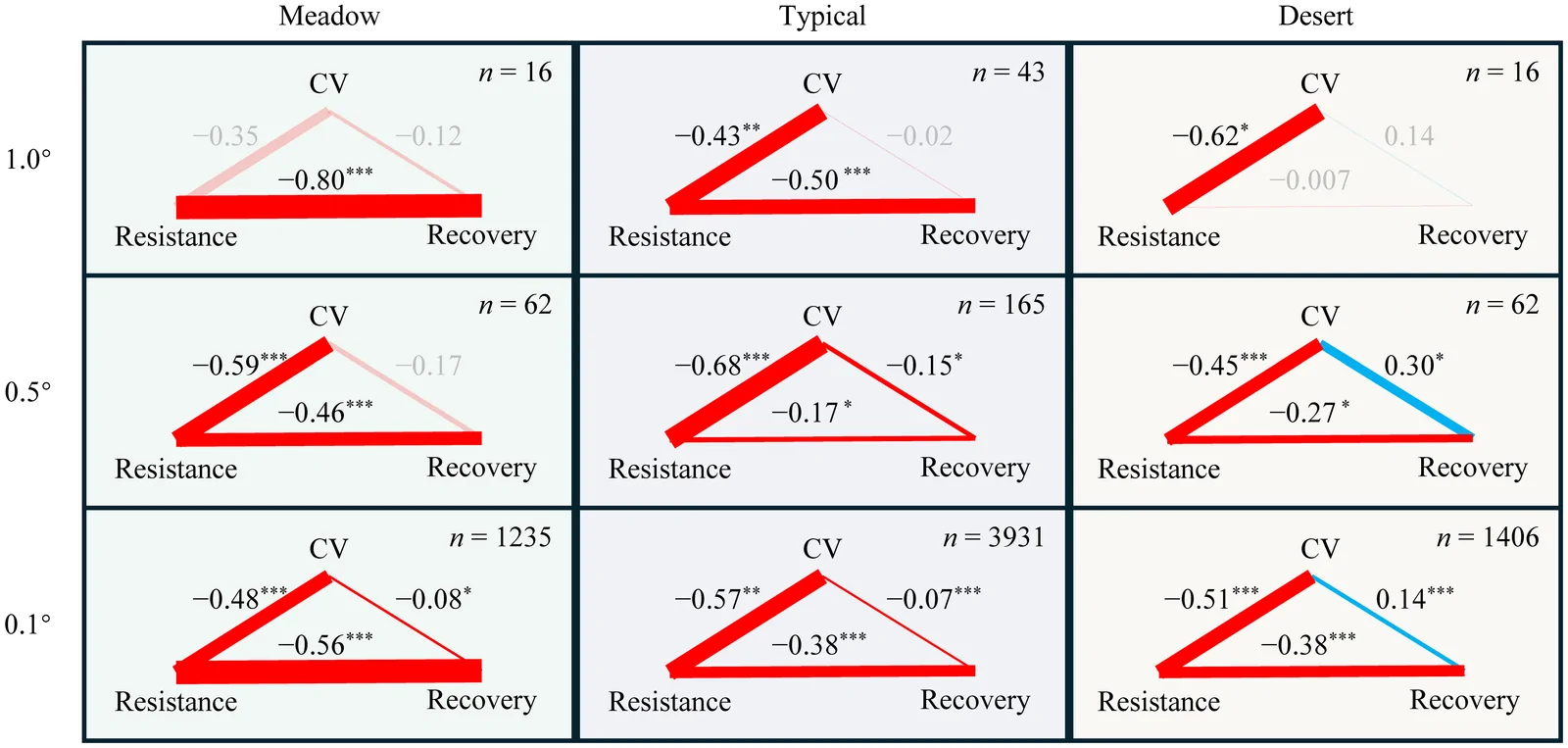
Correlations among local stability dimensions across steppe types and observational grains. Each panel shows Pearson’s correlation coefficients among local community CV, resistance, and recovery for a given steppe type and observational grain. Rows represent observational grains, and columns represent steppe types. Red and blue lines indicate negative and positive correlations, respectively. Saturated line colors represent significant correlations with significance levels denoted as ^*^p < 0.05, ^**^p < 0.01, and ^***^p < 0.001. Faded line colors represent non-significant correlations. Correlation coefficients are displayed along each path, and line thickness is proportional to the magnitude of the correlation. The sample size n represents the number of pixel-year dry events identified for the corresponding steppe type and observational grain. CV, coefficient of variation.

At the local scale, CV and resistance were consistently negatively correlated across all steppe types and observational grains, suggesting that lower CV values (i.e., greater temporal stability) were generally associated with higher resistance (**Figure 3**). In contrast, CV–recovery correlations were weak and variable: meadow and typical steppes showed slight negative associations, whereas desert steppes showed a weak positive association. Resistance and recovery were strongly and consistently negatively correlated regardless of steppe type or observational grain.

### 3.3 Path analysis of factors influencing regional stability across steppe types and observational grains

**Figure 4:**
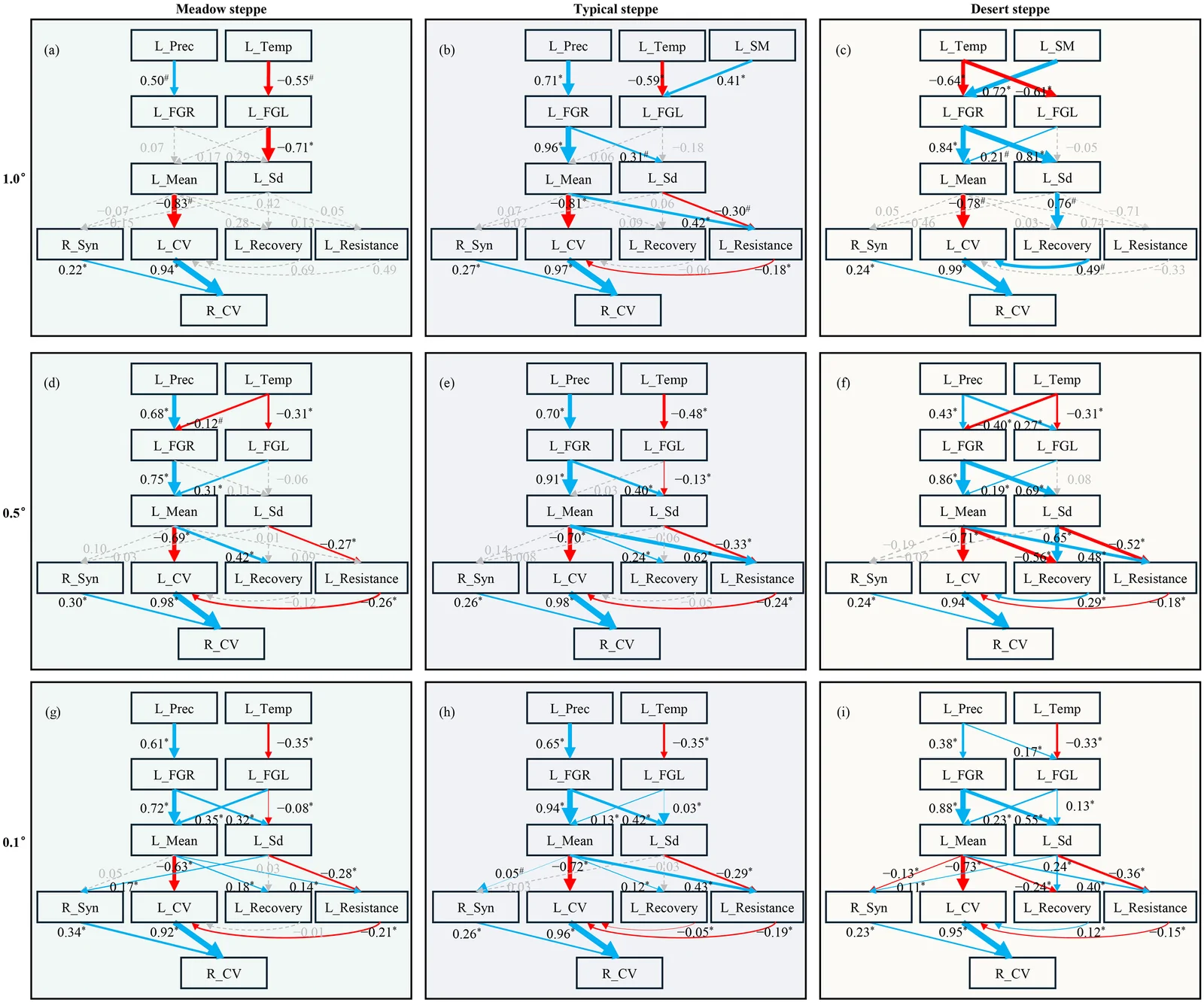
Path analysis models linking local seasonal growth, local stability dimensions, synchrony, and regional temporal variability across steppe types and observational grains. Panels **(a–c)** correspond to meadow, typical, and desert steppes at 1.0°; panels **(d–f)** correspond to meadow, typical, and desert steppes at 0.5°; and panels **(g–i)** correspond to meadow, typical, and desert steppes at 0.1°. Rows represent observational grains, and columns represent steppe types. Numbers beside arrows represent standardized path coefficients estimated from 500 randomized piecewise SEMs. Grey arrows indicate non-robust paths retained to show the hypothesized model structure. Blue and red arrows indicate positive and negative effects, respectively, and line thickness is proportional to the magnitude of the standardized path coefficient. Solid arrows indicate robust paths, and dashed arrows indicate marginally robust paths, based on the proportion of coefficients with signs opposite to the mean coefficient across the 500 randomized SEM runs. The robustness level of each path is indicated by ^*^ when the opposite-sign proportion was< 0.05 and by ^#^ when it was< 0.10. L_Prec, local precipitation; L_Temp, local temperature; L_SM, local soil moisture; L_FGR, local fast-growing phase growth rate; L_FGL, local fast-growing phase length; L_Mean, local mean GPP; L_SD, local GPP standard deviation; R_Syn, regional community synchrony; L_CV, local community coefficient of variation; L_Recovery, local community recovery; L_Resistance, local community resistance; R_CV, regional community coefficient of variation; GPP, gross primary productivity.

Path analysis revealed a clear hierarchical structure in the drivers of regional stability across steppe types and observational grains (**Figure 4**). At the regional scale, regional CV was explained primarily by local CV rather than by spatial synchrony: across all steppe types and grains, the path coefficient from local CV to regional CV consistently exceeded 0.90, whereas the effect of synchrony was much weaker. Nevertheless, the contribution of synchrony showed a grain-dependent gradient at finer grains (0.1°–0.5°), decreasing from meadow to typical to desert steppes.

Across all steppe types and observational grains, higher GPP mean was consistently associated with lower CV, indicating that greater productivity enhanced temporal stability. Resistance showed a similar positive association with GPP mean, with the strongest effects occurring in typical and desert steppes. By contrast, GPP SD was generally negatively associated with resistance, although this relationship was weaker or less robust at the 1.0° grain. Recovery responded to productivity dynamics in a more steppe-specific manner: meadow and typical steppes showed weak positive relationships between recovery and GPP mean, whereas desert steppe exhibited a stronger negative relationship except at the 1.0°. The positive effect of GPP SD on recovery was detected only in desert steppe.

Growth rate was the dominant predictor of GPP mean in most steppe type × grain size combinations. The positive effect of FGR on GPP mean was strongest and most consistent in typical and desert steppes, and remained strong in meadow steppe at 0.5° and 0.1°. By contrast, FGL contributed to mean GPP in several combinations, but its effects were generally weaker and less consistent than those of FGR. The effects of FGR on GPP SD were more variable than those on GPP mean. Higher FGR was positively associated with GPP SD in typical and desert steppes across all observational grains. In meadow steppe, the FGR–GPP SD relationship was positive but was robust only at the 0.1° grain. The effect of FGL on GPP SD was generally weak and inconsistent across steppe types and grains.

Environmental controls on seasonal growth characteristics were clear but context dependent. Precipitation was the dominant positive predictor of FGR across most steppe types and grains, except in desert steppe at 1.0°, where soil moisture exerted a stronger influence. Temperature was retained in all final path models and generally showed negative effects on seasonal growth, with particularly consistent negative effects on FGL and more context-dependent effects on FGR. Overall, favorable moisture conditions promoted faster growth, whereas higher temperature tended to constrain seasonal growth, especially by shortening the fast-growing phase.

## 4 Discussion

Recent cross-continental evidence indicates that Eurasian grasslands are more sensitive to extreme drought than North American grasslands, showing stronger productivity declines under drought stress (Yu et al., 2025). Against this background, Inner Mongolian grasslands, as a major component of the Eurasian steppe, provide an important regional system for examining how drought-sensitive grasslands maintain stability under water limitation. By integrating multidimensional local stability, seasonal growth characteristics, and observational grain, this study clarifies how grassland stability emerges from local to regional scales. Three findings are particularly notable. First, regional stability in Inner Mongolian grasslands was governed primarily by local stability rather than by spatial synchrony. Second, at the local scale, stability was inherently multidimensional, with the relationships among temporal stability, resistance, and recovery varying across steppe types and observational grains. Third, vegetation growth rate during the fast-growing phase (FGR) emerged as a key mechanistic link between environmental conditions and grassland stability, although its effects were pathway-specific and varied across climatic contexts. Together, these findings clarify how grassland stability is organized across scales and show that both climatic context and observational grain need to be considered when interpreting stability and designing management strategies.

### 4.1 Regional stability is governed primarily by local stability rather than spatial synchrony

Regional stability in Inner Mongolian grasslands appears to depend more strongly on local stability than on spatial synchrony among communities (**Figure 4**). Classical spatial insurance theory emphasizes that asynchronous dynamics among local communities can stabilize ecosystem functioning at broader scales by allowing declines in some communities to be offset by gains in others (Loreau and de Mazancourt, 2008; Wang and Loreau, 2014). In our system, however, this stabilizing role of spatial asynchrony seems to be more limited, likely because broad-scale climatic forcing drives local communities to respond in similar directions, thereby reducing the scope for spatial compensation (Tredennick et al., 2017; Wang et al., 2020b). This pattern suggests that the contribution of asynchrony declines from meadow to typical to desert steppes as climatic constraints strengthen and local community responses become more synchronized; accordingly, spatial insurance is likely to remain more important in meadow steppes, where species richness and community heterogeneity are relatively high, than in desert steppes, where simpler community structure and stronger water limitation tend to promote synchronized responses (Wang et al., 2020b; Fu et al., 2022).

For grassland management, this means that efforts to enhance regional stability should not automatically assume that increasing patchiness or spatial heterogeneity is the primary route to greater stability. Heterogeneity may still matter, but in climatically constrained systems, it is not necessarily the primary management priority. Instead, improving the stability of local patches may represent a more direct and potentially effective pathway for enhancing regional stability.

### 4.2 Local stability is multidimensional and varies across steppe types

Local stability in this study was inherently multidimensional: temporal stability, resistance, and recovery were related, but their relationships were not uniform across steppe types and observational grains. Temporal stability was more consistently linked to resistance than to recovery, suggesting that maintaining stable productivity through time in these grasslands depended more strongly on the ability to withstand drought than on post-drought recovery. At the same time, resistance and recovery remained negatively related across contexts, indicating a persistent trade-off between maintaining function during stress and regaining function afterward rather than a single stability axis along which all dimensions improve together (Li et al., 2020; Chen et al., 2023; Yao et al., 2024). This trade-off may occur because communities with high resistance experience smaller functional declines during drought and therefore have less scope for post-drought rebound, whereas communities with low resistance may show stronger recovery once favorable moisture conditions return.

This multidimensional structure differed among steppe types. In meadow and typical steppes, temporal stability was more closely aligned with resistance, whereas recovery played a weaker role. In desert steppe, by contrast, recovery was more closely associated with temporal variability, especially at intermediate grains. This pattern is consistent with the pulse-driven dynamics of arid systems, where opportunistic species can respond quickly to short periods of favorable moisture (Chesson et al., 2004; Zhou et al., 2022). However, rapid recovery does not necessarily lead to greater long-term stability, because post-drought recovery can be incomplete or even overcompensatory, and may therefore be accompanied by greater temporal variability (Chesson et al., 2004; Ru et al., 2023). These patterns suggest that local stability in grasslands should not be interpreted as a single property, but as a combination of stability dimensions whose relative importance shifts across climatic contexts.

A limitation of this drought-event analysis is that the number of pixel-year drought observations was relatively small for meadow steppe and desert steppe at the 1.0° grain. Both combinations had 16 drought observations, and the power analysis indicated limited power to detect moderate correlations at this sample size (**Supplementary Table 14**). Thus, the coarse-grain analyses may have been less able to detect weak or moderate associations among local stability dimensions. Future studies using longer productivity records could increase the number of drought observations and improve the statistical reliability of drought-related stability estimates.

### 4.3 Growth rate provides a key mechanistic link between environmental variation and stability

Our results suggest that growth rate during the fast-growing phase is a key mechanism linking environmental variation to grassland stability. Compared with growth length, growth rate showed a much stronger and more consistent relationship with mean GPP across steppe types and observational grains (**Figure 4**). In Inner Mongolian grasslands, favorable hydrothermal windows are often concentrated within a relatively short portion of the growing season (Piao et al., 2010). Under such conditions, stability may depend less on the length of the growth phase than on how efficiently vegetation converts short favorable hydrothermal windows into productivity (Delgado-Balbuena et al., 2019; Samraoui et al., 2025). Warming during this critical window may weaken this conversion process by increasing evaporative demand, accelerating soil moisture depletion, and reducing photosynthetic carbon gain, thereby limiting the stabilizing effect of fast seasonal growth (Dusenge et al., 2019; Roby et al., 2020).

The effects of growth rate on temporal stability were path-dependent rather than uniformly stabilizing. In most steppe type × grain size combinations, higher FGR was associated with both higher mean GPP and higher GPP SD, indicating that fast seasonal growth increased overall productivity while also enlarging interannual productivity fluctuations. The increase in mean GPP provided a stabilizing pathway by reducing local CV, consistent with the role of average productivity in maintaining temporal stability (Lehman and Tilman, 2000; Isbell et al., 2009). By contrast, the concurrent increase in GPP SD indicates that faster growth was associated with a higher productivity level accompanied by greater year-to-year fluctuations. This pattern is consistent with the resource-use logic of acquisitive growth strategies: rapid growth allows vegetation to capture resources quickly and accumulate biomass under favorable conditions, but it may also make ecosystem productivity more responsive to temporal variation in resource availability (Reich, 2014; Colesie et al., 2020). Notably, the positive effect of FGR on GPP SD became stronger from meadow to typical and desert steppes, suggesting that increasing water limitation may amplify the dependence of productivity on short favorable moisture periods. Nevertheless, because local CV was primarily explained by mean GPP rather than GPP SD in the path models, FGR enhanced temporal stability mainly through its positive effect on mean productivity rather than by reducing productivity variability.

The relationship between FGR and resistance further illustrates this pathway dependence. Across steppe types, higher mean GPP was positively associated with resistance, whereas higher GPP SD was negatively associated with resistance. A higher productivity baseline may help communities maintain ecosystem functioning during drought, while greater interannual productivity fluctuation may indicate stronger dependence on variable resource conditions and a greater likelihood of productivity loss during drought events (Isbell et al., 2015). Thus, the contribution of FGR to resistance depends on whether its positive effect through mean productivity is counteracted by increased productivity variability. Regarding recovery, faster growth can provide the potential for post-drought rebound, but recovery after drought is not determined by growth potential alone. It also depends on the severity of drought-induced productivity loss, the availability of post-drought moisture, and the capacity of vegetation to convert renewed resource supply into biomass gain (Zhou et al., 2022; Ru et al., 2023). In meadow and typical steppes, where resource conditions are relatively less constrained, higher growth rates may facilitate productivity recovery after drought. In desert steppe, however, harsh post-drought conditions and stronger dependence on short-lived resource pulses may limit the realization of this growth potential (Huxman et al., 2004; Reynolds et al., 2004).

Taken together, these patterns are consistent with the resource-use logic of the plant economics spectrum, in which faster growth reflects a more acquisitive capacity to capture resources and accumulate biomass under favorable conditions (Reich, 2014). Extending this logic to ecosystem level, our results suggest that rapid seasonal growth can enhance mean productivity and recovery potential, but its contribution to temporal stability, resistance, and recovery depends on how this growth potential is expressed under local climatic constraints.

These interpretations should also be viewed in light of potential spatial autocorrelation, because neighboring pixels within the same steppe type may share similar climatic conditions and GPP dynamics. Future studies could use spatially explicit models or spatial block-resampling approaches to further test the robustness of these pathways. From a management perspective, these findings suggest that maintaining grassland stability requires attention to the critical fast-growing phase rather than relying only on annual averages of grazing pressure or environmental conditions. Protecting vegetation and reducing additional stress during this window may be especially important for sustaining productivity and stability under increasing climatic variability.

### 4.4 Observational grain modifies the expression and interpretation of stability

Observational grain in this study was not simply a technical property of the data, but an important factor shaping how stability was expressed and interpreted. Coarser observational grains tended to exhibit higher temporal stability because spatial aggregation averaged local heterogeneity and dampened fine-scale variability. By contrast, finer grains retained greater local stochasticity and small-scale fluctuations (Jelinski and Wu, 1996; Wang and Loreau, 2014; Liang et al., 2025). This aggregation effect may also explain why post-drought recovery showed different rankings among steppe types across grains. Recovery depends not only on how drought-induced productivity losses but also how subsequent rebounds are spatially integrated across heterogeneous patches (Wang et al., 2019; Zhang et al., 2023). At the 0.5° grain, typical steppe may better retain intermediate-scale heterogeneity in productivity responses, allowing rapidly recovering patches to compensate for more strongly affected patches. By contrast, at the 1.0° grain, broader aggregation may smooth local post-drought deviations and emphasize the stronger landscape-level recovery potential of the relatively moist and productive meadow steppe.

Moreover, grain influenced not only the absolute values of stability metrics, but also how clearly relationships among stability dimensions could be detected. In our results, the intermediate grain provided the clearest links among temporal stability, resistance, and recovery, possibly because it reduced fine-scale stochasticity without fully erasing spatial heterogeneity. These results make clear that stability-oriented management must be explicit about spatial scale. Conclusions drawn from fine-grain observations cannot be directly extended to broader management units, just as patterns observed at coarse grains may overlook local processes that are important for intervention. In practice, this means that the spatial scale at which stability is evaluated should match the spatial scale at which management is intended to operate. For grassland management, the relevant question is therefore not only how stable the system is, but also at what spatial grain that stability is being assessed and targeted.

## 5 Conclusions

This study shows that regional stability in Inner Mongolian grasslands is maintained primarily through local stability rather than spatial synchrony among communities. At the local scale, temporal stability, resistance, and recovery represent distinct dimensions of stability, and their relationships vary across steppe types and observational grains. FGR provides a key mechanistic link between environmental variation and stability by reflecting how efficiently vegetation converts favorable hydrothermal conditions into productivity during the fast-growing phase. It strengthens temporal stability mainly by increasing mean GPP, while its effects on resistance and recovery depend on the balance between productivity level, productivity variability, and post-drought resource conditions. Overall, favorable hydrothermal conditions during the critical fast-growing phase can promote FGR, strengthen local stability, and thereby enhance regional stability, but the stabilizing role of FGR is pathway-specific rather than universal. For management, protecting vegetation and reducing additional stress during this critical growth window are important, while management measures should also be matched to the observational grain and spatial scale at which stability is assessed and targeted.

## Data Availability

The original contributions presented in the study are included in the article/**Supplementary Material**. Further inquiries can be directed to the corresponding authors.
