# Correction: Fast seasonal growth promotes regional stability by strengthening local temporal stability in Inner Mongolian grasslands

**DOI:** 10.3389/fpls.2026.1954864

**Published:** 2026-09-07

**Authors:** Frontiers Production Office

**Affiliations:** Frontiers Media SA, Lausanne, Switzerland

**Keywords:** climate change, grassland, multidimensional stability, regional stability, seasonal growth characteristics

There was a mistake in **Figure 3** as published. In the top-right panel (Desert, 1.0°), the label “Invariability” was mistakenly used. The correct label should be “CV”, consistent with the other panels in the figure. The corrected **Figure 3** appears below.

**Figure 3:**
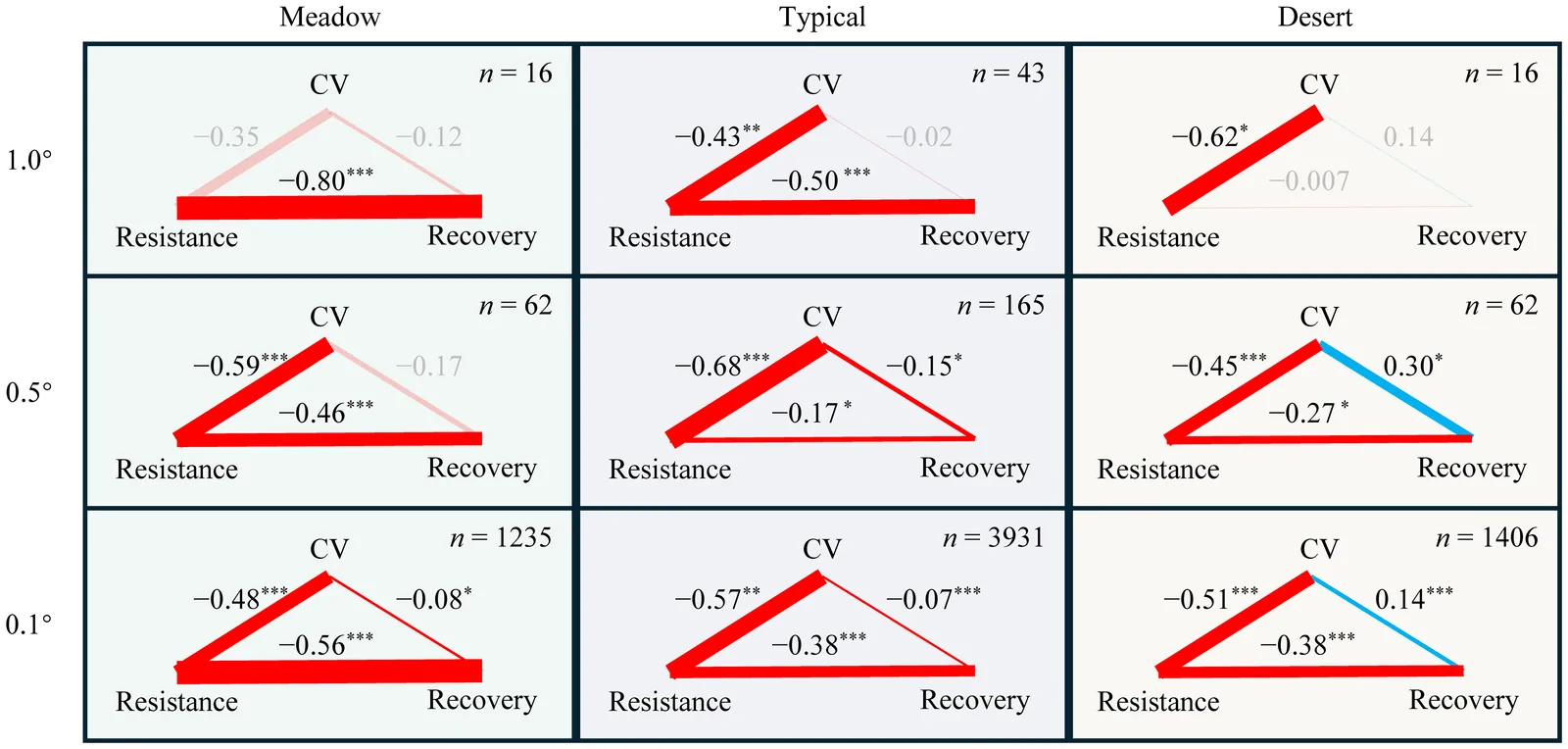
Correlations among local stability dimensions across steppe types and observational grains. Each panel shows Pearson’s correlation coefficients among local community CV, resistance, and recovery for a given steppe type and observational grain. Rows represent observational grains, and columns represent steppe types. Red and blue lines indicate negative and positive correlations, respectively. Saturated line colors represent significant correlations with significance levels denoted as *p < 0.05, **p < 0.01, and ***p < 0.001. Faded line colors represent non-significant correlations. Correlation coefficients are displayed along each path, and line thickness is proportional to the magnitude of the correlation. The sample size n represents the number of pixel-year dry events identified for the corresponding steppe type and observational grain. CV, coefficient of variation.

The authors apologize for this error and state that this does not change the scientific conclusions of the article in any way.

The original version of this article has been updated.

