# Supplementary material for: Fast seasonal growth promotes regional stability by strengthening local temporal stability in Inner Mongolian grasslands
Source: Front Plant Sci. 2026 Jul 23;17:1902582. doi: 10.3389/fpls.2026.1902582 (PMC13444368; doi:10.3389/fpls.2026.1902582)
Supplement: Supplementary file 1 [file DataSheet1.docx]

Supplementary Material

**Fast seasonal growth promotes regional stability by strengthening local temporal stability in Inner Mongolian grasslands**

**Zexu Nie^1^,Yujie Yuan^1^, Hushun Zhang^1^, Fang Shi^1^, Han Zhang^2,*^, Chao Wang^3,*^**

^1^Key Laboratory of Ecological Impacts of Hydraulic-Projects and Restoration of Aquatic Ecosystem of Ministry of Water Resources, Institute of Hydroecology, Ministry of Water Resources and Chinese Academy of Sciences, Wuhan, China

^2^School of Urban Design, Wuhan University, Wuhan, China

^3^National Permanent Scientific Research Base for Warm Temperate Zone Forestry of Jiulong Mountain in Beijing, Experimental Center of Forestry in North China, Chinese Academy of Forestry, Beijing 102300, China

***Corresponding author:**

Han Zhang

E-mail: [zhanghan_2000@whu.edu.cn](mailto:zhanghan_2000@whu.edu.cn)

Chao Wang

E-mail: [ecowangch13@gmail.com](mailto:ecowangch13@gmail.com)

## **Supplementary Tables**

Table S1 Drought-observation sample size and AIC distributions of final path models

| Steppe type | Grain size | Number of pixel-year drought observations | AIC_Mean | AIC_2.5 | AIC_97.5 |
| --- | --- | --- | --- | --- | --- |
| Meadow steppe | 0.1° | 1235 | 10698 | 10474 | 10937 |
|  | 0.5° | 62 | 417 | 366 | 464 |
|  | 1.0° | 16 | 94 | 45 | 130 |
| Typical steppe | 0.1° | 3931 | 37995 | 37562 | 38434 |
|  | 0.5° | 165 | 1416 | 1326 | 1498 |
|  | 1.0° | 43 | 318 | 266 | 360 |
| Desert steppe | 0.1° | 1406 | 8523 | 8273 | 8751 |
|  | 0.5° | 62 | 215 | 167 | 262 |
|  | 1.0° | 16 | 37 | 7 | 70 |

Note: Number of pixel-year drought observations indicates the number of pixel-year observations identified as drought events for each steppe type and grain size. AIC values were calculated for the final path models across the 500 randomized model runs. AIC_Mean represents the mean AIC, while AIC_2.5 and AIC_97.5 represent the 2.5th and 97.5th percentiles of the AIC distribution, respectively.

Table S2. Standardized path coefficients for meadow steppe at the 1.0° grain

| Path | Mean coefficient | Median coefficient | Coefficient 2.5% | Coefficient 97.5% | Sig |
| --- | --- | --- | --- | --- | --- |
| R_CV<-L_CV | 0.94 | 0.93 | 0.75 | 1.14 | * |
| R_CV<-R_Syn | 0.22 | 0.20 | 0.09 | 0.45 | * |
| R_Syn<-L_Mean | -0.07 | -0.13 | -0.99 | 0.93 | ns |
| R_Syn<-L_SD | 0.15 | 0.2 | -0.83 | 1.06 | ns |
| L_CV<-L_Mean | -0.83 | -0.84 | -3.16 | 1.37 | # |
| L_CV<-L_Resistance | 0.49 | 0.3 | -1.72 | 4.43 | ns |
| L_CV<-L_Recovery | 0.69 | 0.45 | -1.39 | 4.38 | ns |
| L_Resistance<-L_Mean | 0.13 | 0.23 | -1.21 | 1.11 | ns |
| L_Resistance<-L_SD | 0.05 | 0.09 | -1.14 | 1.03 | ns |
| L_Recovery<-L_Mean | 0.28 | 0.29 | -0.92 | 1.7 | ns |
| L_Recovery<-L_SD | 0.42 | 0.39 | -0.76 | 1.57 | ns |
| L_Mean<-L_FGR | 0.07 | 0.16 | -1.04 | 1.26 | ns |
| L_Mean<-L_FGL | 0.17 | 0.28 | -1.08 | 1.30 | ns |
| L_SD<-L_FGR | 0.29 | 0.35 | -0.61 | 0.88 | ns |
| L_SD<-L_FGL | -0.71 | -0.75 | -1.24 | 0.02 | * |
| L_FGR<-L_Prec | 0.50 | 0.62 | -0.68 | 0.93 | ns |
| L_FGL<-L_Temp | -0.55 | -0.60 | -0.93 | 0.20 | # |

Note: Values are standardized path coefficients estimated from 500 randomized model runs. “Mean coefficient” represents the mean standardized coefficient across the 500 runs, “Median coefficient” represents the median standardized coefficient, and “Coefficient 2.5%” and “Coefficient 97.5%” represent the 2.5th and 97.5th percentiles of the coefficient distribution, respectively. Sig. indicates the robustness of path direction across the 500 randomized model runs: * indicates that the proportion of coefficients with signs opposite to the mean coefficient was < 0.05; # indicates that this proportion was < 0.10; ns indicates this proportion was > 0.10. L_CV: local community coefficient of variation; R_CV: regional community coefficient of variation; R_Syn: regional community synchrony; L_Mean: local mean GPP; L_SD: local GPP standard deviation; L_Resistance: local community resistance; L_Recovery: local community recovery; L_FGR: local fast-growing phase growth rate; L_FGL: local fast-growing phase length; L_Prec: local precipitation; L_Temp: local temperature.

Table S3. Standardized path coefficients for typical steppe at the 1.0° grain

| Path | Mean coefficient | Median coefficient | Coefficient 2.5% | Coefficient 97.5% | Sig |
| --- | --- | --- | --- | --- | --- |
| R_CV<-L_CV | 0.97 | 0.97 | 0.86 | 1.08 | * |
| R_CV<-R_Syn | 0.27 | 0.26 | 0.19 | 0.37 | * |
| R_Syn<-L_Mean | 0.07 | 0.08 | -0.42 | 0.55 | ns |
| R_Syn<-L_SD | 0.02 | 0.01 | -0.42 | 0.50 | ns |
| L_CV<-L_Mean | -0.81 | -0.81 | -0.93 | -0.64 | * |
| L_CV<-L_Resistance | -0.18 | -0.18 | -0.39 | 0.03 | # |
| L_CV<-L_Recovery | -0.06 | -0.06 | -0.26 | 0.14 | ns |
| L_Resistance<-L_Mean | 0.42 | 0.41 | 0.09 | 0.79 | * |
| L_Resistance<-L_SD | -0.30 | -0.31 | -0.65 | 0.09 | # |
| L_Recovery<-L_Mean | 0.09 | 0.08 | -0.28 | 0.46 | ns |
| L_Recovery<-L_SD | 0.06 | 0.06 | -0.29 | 0.45 | ns |
| L_Mean<-L_FGR | 0.96 | 0.96 | 0.82 | 1.07 | * |
| L_Mean<-L_FGL | 0.06 | 0.06 | -0.13 | 0.24 | ns |
| L_SD<-L_FGR | 0.31 | 0.32 | -0.15 | 0.61 | # |
| L_SD<-L_FGL | -0.18 | -0.18 | -0.54 | 0.19 | ns |
| L_FGR<-L_Prec | 0.71 | 0.72 | 0.47 | 0.87 | * |
| L_FGL<-L_Temp | -0.59 | -0.59 | -0.79 | -0.35 | * |
| L_FGL<-L_SM | 0.41 | 0.4 | 0.14 | 0.67 | * |

Note: Values are standardized path coefficients estimated from 500 randomized model runs. “Mean coefficient” represents the mean standardized coefficient across the 500 runs, “Median coefficient” represents the median standardized coefficient, and “Coefficient 2.5%” and “Coefficient 97.5%” represent the 2.5th and 97.5th percentiles of the coefficient distribution, respectively. Sig. indicates the robustness of path direction across the 500 randomized model runs: * indicates that the proportion of coefficients with signs opposite to the mean coefficient was < 0.05; # indicates that this proportion was < 0.10; ns indicates this proportion was > 0.10. L_CV: local community coefficient of variation; R_CV: regional community coefficient of variation; R_Syn: regional community synchrony; L_Mean: local mean GPP; L_SD: local GPP standard deviation; L_Resistance: local community resistance; L_Recovery: local community recovery; L_FGR: local fast-growing phase growth rate; L_FGL: local fast-growing phase length; L_Prec: local precipitation; L_Temp: local temperature; L_SM: local soil moisture;GPP: gross primary productivity.

Table S4. Standardized path coefficients for desert steppe at the 1.0° grain

| Path | Mean coefficient | Median coefficient | Coefficient 2.5% | Coefficient 97.5% | Sig |
| --- | --- | --- | --- | --- | --- |
| R_CV<-L_CV | 0.99 | 0.98 | 0.81 | 1.20 | * |
| R_CV<-R_Syn | 0.24 | 0.23 | 0.11 | 0.47 | * |
| R_Syn<-L_Mean | 0.05 | 0.07 | -1.52 | 1.39 | ns |
| R_Syn<-L_SD | -0.46 | -0.57 | -1.61 | 1.26 | ns |
| L_CV<-L_Mean | -0.78 | -0.82 | -1.79 | 0.27 | # |
| L_CV<-L_Resistance | -0.33 | -0.32 | -1.07 | 0.31 | ns |
| L_CV<-L_Recovery | 0.49 | 0.49 | -0.37 | 1.23 | # |
| L_Resistance<-L_Mean | 0.74 | 0.77 | -0.78 | 2.12 | ns |
| L_Resistance<-L_SD | -0.71 | -0.73 | -1.93 | 0.65 | ns |
| L_Recovery<-L_Mean | -0.03 | -0.08 | -0.77 | 0.99 | ns |
| L_Recovery<-L_SD | 0.77 | 0.84 | -0.22 | 1.40 | # |
| L_Mean<-L_FGR | 0.84 | 0.87 | 0.50 | 0.99 | * |
| L_Mean<-L_FGL | 0.21 | 0.19 | -0.07 | 0.54 | # |
| L_SD<-L_FGR | 0.81 | 0.82 | 0.24 | 1.27 | * |
| L_SD<-L_FGL | -0.05 | -0.02 | -0.62 | 0.59 | ns |
| L_FGR<-L_Temp | -0.64 | -0.63 | -1.40 | 0.04 | * |
| L_FGR<-L_SM | 0.72 | 0.75 | -0.16 | 1.50 | # |
| L_FGL<-L_Temp | -0.61 | -0.68 | -0.94 | 0.16 | # |

Note: Values are standardized path coefficients estimated from 500 randomized model runs. “Mean coefficient” represents the mean standardized coefficient across the 500 runs, “Median coefficient” represents the median standardized coefficient, and “Coefficient 2.5%” and “Coefficient 97.5%” represent the 2.5th and 97.5th percentiles of the coefficient distribution, respectively. Sig. indicates the robustness of path direction across the 500 randomized model runs: * indicates that the proportion of coefficients with signs opposite to the mean coefficient was < 0.05; # indicates that this proportion was < 0.10; ns indicates this proportion was > 0.10. L_CV: local community coefficient of variation; R_CV: regional community coefficient of variation; R_Syn: regional community synchrony; L_Mean: local mean GPP; L_SD: local GPP standard deviation; L_Resistance: local community resistance; L_Recovery: local community recovery; L_FGR: local fast-growing phase growth rate; L_FGL: local fast-growing phase length; L_Prec: local precipitation; L_Temp: local temperature; L_SM: local soil moisture;GPP: gross primary productivity.

Table S5. Standardized path coefficients for meadow steppe at the 0.5° grain

| Path | Mean coefficient | Median coefficient | Coefficient 2.5% | Coefficient 97.5% | Sig |
| --- | --- | --- | --- | --- | --- |
| R_CV<-L_CV | 0.98 | 0.98 | 0.88 | 1.11 | * |
| R_CV<-R_Syn | 0.30 | 0.29 | 0.18 | 0.46 | * |
| R_Syn<-L_Mean | 0.10 | 0.10 | -0.30 | 0.48 | ns |
| R_Syn<-L_SD | -0.03 | -0.03 | -0.4 | 0.35 | ns |
| L_CV<-L_Mean | -0.69 | -0.69 | -0.89 | -0.50 | * |
| L_CV<-L_Resistance | -0.26 | -0.26 | -0.51 | -0.01 | * |
| L_CV<-L_Recovery | -0.12 | -0.12 | -0.35 | 0.14 | ns |
| L_Resistance<-L_Mean | 0.09 | 0.11 | -0.28 | 0.40 | ns |
| L_Resistance<-L_SD | -0.27 | -0.28 | -0.53 | 0.02 | * |
| L_Recovery<-L_Mean | 0.42 | 0.43 | 0.08 | 0.67 | * |
| L_Recovery<-L_SD | 0.01 | 0.01 | -0.25 | 0.26 | ns |
| L_Mean<-L_FGR | 0.75 | 0.76 | 0.59 | 0.87 | * |
| L_Mean<-L_FGL | 0.31 | 0.32 | 0.12 | 0.49 | * |
| L_SD<-L_FGR | 0.11 | 0.10 | -0.17 | 0.40 | ns |
| L_SD<-L_FGL | -0.06 | -0.06 | -0.30 | 0.21 | ns |
| L_FGR<-L_Prec | 0.69 | 0.70 | 0.45 | 0.83 | * |
| L_FGR<-L_Temp | -0.12 | -0.12 | -0.33 | 0.07 | # |
| L_FGL<-L_Prec | -0.04 | -0.04 | -0.34 | 0.29 | ns |
| L_FGL<-L_Temp | -0.31 | -0.32 | -0.56 | 0.01 | * |

Note: Values are standardized path coefficients estimated from 500 randomized model runs. “Mean coefficient” represents the mean standardized coefficient across the 500 runs, “Median coefficient” represents the median standardized coefficient, and “Coefficient 2.5%” and “Coefficient 97.5%” represent the 2.5th and 97.5th percentiles of the coefficient distribution, respectively. Sig. indicates the robustness of path direction across the 500 randomized model runs: * indicates that the proportion of coefficients with signs opposite to the mean coefficient was < 0.05; # indicates that this proportion was < 0.10; ns indicates this proportion was > 0.10. L_CV: local community coefficient of variation; R_CV: regional community coefficient of variation; R_Syn: regional community synchrony; L_Mean: local mean GPP; L_SD: local GPP standard deviation; L_Resistance: local community resistance; L_Recovery: local community recovery; L_FGR: local fast-growing phase growth rate; L_FGL: local fast-growing phase length; L_Prec: local precipitation; L_Temp: local temperature.

Table S6. Standardized path coefficients for typical steppe at the 0.5° grain

| Path | Mean coefficient | Median coefficient | Coefficient 2.5% | Coefficient 97.5% | Sig |
| --- | --- | --- | --- | --- | --- |
| R_CV<-L_CV | 0.98 | 0.98 | 0.93 | 1.03 | * |
| R_CV<-R_Syn | 0.26 | 0.25 | 0.22 | 0.30 | * |
| R_Syn<-L_Mean | 0.14 | 0.14 | -0.09 | 0.37 | ns |
| R_Syn<-L_SD | 0.01 | 0.01 | -0.25 | 0.24 | ns |
| L_CV<-L_Mean | -0.7 | -0.71 | -0.79 | -0.61 | * |
| L_CV<-L_Resistance | -0.24 | -0.24 | -0.33 | -0.14 | * |
| L_CV<-L_Recovery | -0.05 | -0.05 | -0.14 | 0.04 | ns |
| L_Resistance<-L_Mean | 0.62 | 0.62 | 0.46 | 0.77 | * |
| L_Resistance<-L_SD | -0.33 | -0.33 | -0.49 | -0.18 | * |
| L_Recovery<-L_Mean | 0.24 | 0.25 | 0.08 | 0.42 | * |
| L_Recovery<-L_SD | -0.06 | -0.06 | -0.23 | 0.11 | ns |
| L_Mean<-L_FGR | 0.91 | 0.91 | 0.86 | 0.95 | * |
| L_Mean<-L_FGL | 0.03 | 0.03 | -0.06 | 0.11 | ns |
| L_SD<-L_FGR | 0.4 | 0.40 | 0.26 | 0.55 | * |
| L_SD<-L_FGL | -0.13 | -0.13 | -0.27 | 0.02 | # |
| L_FGR<-L_Prec | 0.70 | 0.70 | 0.59 | 0.79 | * |
| L_FGL<-L_Temp | -0.48 | -0.48 | -0.60 | -0.35 | * |

Note: Values are standardized path coefficients estimated from 500 randomized model runs. “Mean coefficient” represents the mean standardized coefficient across the 500 runs, “Median coefficient” represents the median standardized coefficient, and “Coefficient 2.5%” and “Coefficient 97.5%” represent the 2.5th and 97.5th percentiles of the coefficient distribution, respectively. Sig. indicates the robustness of path direction across the 500 randomized model runs: * indicates that the proportion of coefficients with signs opposite to the mean coefficient was < 0.05; # indicates that this proportion was < 0.10; ns indicates this proportion was > 0.10. L_CV: local community coefficient of variation; R_CV: regional community coefficient of variation; R_Syn: regional community synchrony; L_Mean: local mean GPP; L_SD: local GPP standard deviation; L_Resistance: local community resistance; L_Recovery: local community recovery; L_FGR: local fast-growing phase growth rate; L_FGL: local fast-growing phase length; L_Prec: local precipitation; L_Temp: local temperature.

Table S7. Standardized path coefficients for desert steppe at the 0.5° grain

| Path | Mean coefficient | Median coefficient | Coefficient 2.5% | Coefficient 97.5% | Sig |
| --- | --- | --- | --- | --- | --- |
| R_CV<-L_CV | 0.94 | 0.94 | 0.84 | 1.05 | * |
| R_CV<-R_Syn | 0.24 | 0.23 | 0.17 | 0.31 | * |
| R_Syn<-L_Mean | -0.19 | -0.22 | -0.86 | 0.67 | ns |
| R_Syn<-L_SD | -0.02 | -0.01 | -0.72 | 0.73 | ns |
| L_CV<-L_Mean | -0.71 | -0.71 | -0.84 | -0.57 | * |
| L_CV<-L_Resistance | -0.18 | -0.18 | -0.37 | 0.04 | * |
| L_CV<-L_Recovery | 0.29 | 0.30 | 0.08 | 0.49 | * |
| L_Resistance<-L_Mean | 0.48 | 0.48 | 0.01 | 0.92 | * |
| L_Resistance<-L_SD | -0.52 | -0.54 | -0.98 | 0.01 | * |
| L_Recovery<-L_Mean | -0.56 | -0.57 | -1.05 | -0.03 | * |
| L_Recovery<-L_SD | 0.65 | 0.64 | 0.14 | 1.15 | * |
| L_Mean<-L_FGR | 0.86 | 0.87 | 0.7 | 0.96 | * |
| L_Mean<-L_FGL | 0.19 | 0.19 | 0.01 | 0.36 | * |
| L_SD<-L_FGR | 0.69 | 0.69 | 0.45 | 0.87 | * |
| L_SD<-L_FGL | 0.08 | 0.08 | -0.17 | 0.33 | ns |
| L_FGR<-L_Prec | 0.43 | 0.43 | 0.16 | 0.68 | * |
| L_FGR<-L_Temp | -0.40 | -0.42 | -0.60 | -0.14 | * |
| L_FGL<-L_Prec | 0.27 | 0.28 | -0.09 | 0.58 | * |
| L_FGL<-L_Temp | -0.31 | -0.33 | -0.58 | 0.01 | * |

Note: Values are standardized path coefficients estimated from 500 randomized model runs. “Mean coefficient” represents the mean standardized coefficient across the 500 runs, “Median coefficient” represents the median standardized coefficient, and “Coefficient 2.5%” and “Coefficient 97.5%” represent the 2.5th and 97.5th percentiles of the coefficient distribution, respectively. Sig. indicates the robustness of path direction across the 500 randomized model runs: * indicates that the proportion of coefficients with signs opposite to the mean coefficient was < 0.05; # indicates that this proportion was < 0.10; ns indicates this proportion was > 0.10. L_CV: local community coefficient of variation; R_CV: regional community coefficient of variation; R_Syn: regional community synchrony; L_Mean: local mean GPP; L_SD: local GPP standard deviation; L_Resistance: local community resistance; L_Recovery: local community recovery; L_FGR: local fast-growing phase growth rate; L_FGL: local fast-growing phase length; L_Prec: local precipitation; L_Temp: local temperature.

Table S8. Standardized path coefficients for meadow steppe at the 0.1° grain

| Path | Mean coefficient | Median coefficient | Coefficient 2.5% | Coefficient 97.5% | Sig |
| --- | --- | --- | --- | --- | --- |
| R_CV<-L_CV | 0.92 | 0.92 | 0.90 | 0.94 | * |
| R_CV<-R_Syn | 0.34 | 0.34 | 0.32 | 0.37 | * |
| R_Syn<-L_Mean | 0.05 | 0.05 | -0.03 | 0.14 | ns |
| R_Syn<-L_SD | 0.17 | 0.17 | 0.08 | 0.25 | * |
| L_CV<-L_Mean | -0.63 | -0.63 | -0.66 | -0.6 | * |
| L_CV<-L_Resistance | -0.21 | -0.21 | -0.25 | -0.16 | * |
| L_CV<-L_Recovery | -0.01 | -0.01 | -0.06 | 0.03 | ns |
| L_Resistance<-L_Mean | 0.14 | 0.14 | 0.08 | 0.20 | * |
| L_Resistance<-L_SD | -0.28 | -0.28 | -0.33 | -0.22 | * |
| L_Recovery<-L_Mean | 0.18 | 0.17 | 0.12 | 0.24 | * |
| L_Recovery<-L_SD | 0.03 | 0.03 | -0.02 | 0.09 | ns |
| L_Mean<-L_FGR | 0.72 | 0.72 | 0.69 | 0.75 | * |
| L_Mean<-L_FGL | 0.35 | 0.35 | 0.32 | 0.39 | * |
| L_SD<-L_FGR | 0.32 | 0.32 | 0.26 | 0.37 | * |
| L_SD<-L_FGL | -0.08 | -0.08 | -0.13 | -0.02 | * |
| L_FGR<-L_Prec | 0.61 | 0.61 | 0.56 | 0.65 | * |
| L_FGR<-L_Temp | -0.07 | -0.07 | -0.12 | -0.03 | * |
| L_FGL<-L_Prec | -0.08 | -0.08 | -0.14 | -0.03 | * |
| L_FGL<-L_Temp | -0.35 | -0.36 | -0.4 | -0.3 | * |

Note: Values are standardized path coefficients estimated from 500 randomized model runs. “Mean coefficient” represents the mean standardized coefficient across the 500 runs, “Median coefficient” represents the median standardized coefficient, and “Coefficient 2.5%” and “Coefficient 97.5%” represent the 2.5th and 97.5th percentiles of the coefficient distribution, respectively. Sig. indicates the robustness of path direction across the 500 randomized model runs: * indicates that the proportion of coefficients with signs opposite to the mean coefficient was < 0.05; # indicates that this proportion was < 0.10; ns indicates this proportion was > 0.10. L_CV: local community coefficient of variation; R_CV: regional community coefficient of variation; R_Syn: regional community synchrony; L_Mean: local mean GPP; L_SD: local GPP standard deviation; L_Resistance: local community resistance; L_Recovery: local community recovery; L_FGR: local fast-growing phase growth rate; L_FGL: local fast-growing phase length; L_Prec: local precipitation; L_Temp: local temperature.

Table S9. Standardized path coefficients for typical steppe at the 0.1° grain

| Path | Mean coefficient | Median coefficient | Coefficient 2.5% | Coefficient 97.5% | Sig |
| --- | --- | --- | --- | --- | --- |
| R_CV<-L_CV | 0.96 | 0.96 | 0.95 | 0.97 | * |
| R_CV<-R_Syn | 0.26 | 0.26 | 0.25 | 0.27 | * |
| R_Syn<-L_Mean | 0.05 | 0.06 | 0.01 | 0.11 | * |
| R_Syn<-L_SD | 0.03 | 0.03 | -0.02 | 0.08 | ns |
| L_CV<-L_Mean | -0.72 | -0.72 | -0.74 | -0.71 | * |
| L_CV<-L_Resistance | -0.19 | -0.19 | -0.21 | -0.16 | * |
| L_CV<-L_Recovery | -0.05 | -0.05 | -0.07 | -0.03 | * |
| L_Resistance<-L_Mean | 0.43 | 0.43 | 0.4 | 0.46 | * |
| L_Resistance<-L_SD | -0.29 | -0.29 | -0.32 | -0.25 | * |
| L_Recovery<-L_Mean | 0.12 | 0.12 | 0.09 | 0.16 | * |
| L_Recovery<-L_SD | -0.03 | -0.03 | -0.06 | 0.01 | # |
| L_Mean<-L_FGR | 0.94 | 0.94 | 0.93 | 0.95 | * |
| L_Mean<-L_FGL | 0.13 | 0.13 | 0.11 | 0.15 | * |
| L_SD<-L_FGR | 0.42 | 0.42 | 0.39 | 0.45 | * |
| L_SD<-L_FGL | 0.03 | 0.03 | 0 | 0.06 | * |
| L_FGR<-L_Prec | 0.65 | 0.65 | 0.63 | 0.67 | * |
| L_FGL<-L_Temp | -0.35 | -0.35 | -0.38 | -0.33 | * |

Note: Values are standardized path coefficients estimated from 500 randomized model runs. “Mean coefficient” represents the mean standardized coefficient across the 500 runs, “Median coefficient” represents the median standardized coefficient, and “Coefficient 2.5%” and “Coefficient 97.5%” represent the 2.5th and 97.5th percentiles of the coefficient distribution, respectively. Sig. indicates the robustness of path direction across the 500 randomized model runs: * indicates that the proportion of coefficients with signs opposite to the mean coefficient was < 0.05; # indicates that this proportion was < 0.10; ns indicates this proportion was > 0.10. L_CV: local community coefficient of variation; R_CV: regional community coefficient of variation; R_Syn: regional community synchrony; L_Mean: local mean GPP; L_SD: local GPP standard deviation; L_Resistance: local community resistance; L_Recovery: local community recovery; L_FGR: local fast-growing phase growth rate; L_FGL: local fast-growing phase length; L_Prec: local precipitation; L_Temp: local temperature.

Table S10. Standardized path coefficients for desert steppe at the 0.1° grain

| Path | Mean coefficient | Median coefficient | Coefficient 2.5% | Coefficient 97.5% | Sig |
| --- | --- | --- | --- | --- | --- |
| R_CV<-L_CV | 0.95 | 0.95 | 0.93 | 0.97 | * |
| R_CV<-R_Syn | 0.23 | 0.23 | 0.22 | 0.24 | * |
| R_Syn<-L_Mean | -0.13 | -0.13 | -0.23 | -0.02 | * |
| R_Syn<-L_SD | 0.11 | 0.11 | -0.01 | 0.21 | * |
| L_CV<-L_Mean | -0.73 | -0.73 | -0.76 | -0.71 | * |
| L_CV<-L_Resistance | -0.15 | -0.15 | -0.19 | -0.11 | * |
| L_CV<-L_Recovery | 0.12 | 0.12 | 0.08 | 0.16 | * |
| L_Resistance<-L_Mean | 0.4 | 0.40 | 0.32 | 0.47 | * |
| L_Resistance<-L_SD | -0.36 | -0.36 | -0.43 | -0.29 | * |
| L_Recovery<-L_Mean | -0.24 | -0.24 | -0.31 | -0.16 | * |
| L_Recovery<-L_SD | 0.24 | 0.24 | 0.15 | 0.32 | * |
| L_Mean<-L_FGR | 0.88 | 0.88 | 0.86 | 0.9 | * |
| L_Mean<-L_FGL | 0.23 | 0.23 | 0.19 | 0.26 | * |
| L_SD<-L_FGR | 0.55 | 0.55 | 0.5 | 0.59 | * |
| L_SD<-L_FGL | 0.13 | 0.12 | 0.07 | 0.18 | * |
| L_FGR<-L_Prec | 0.38 | 0.38 | 0.33 | 0.44 | * |
| L_FGL<-L_Prec | 0.17 | 0.17 | 0.11 | 0.22 | * |
| L_FGL<-L_Temp | -0.33 | -0.34 | -0.39 | -0.28 | * |

Note: Values are standardized path coefficients estimated from 500 randomized model runs. “Mean coefficient” represents the mean standardized coefficient across the 500 runs, “Median coefficient” represents the median standardized coefficient, and “Coefficient 2.5%” and “Coefficient 97.5%” represent the 2.5th and 97.5th percentiles of the coefficient distribution, respectively. Sig. indicates the robustness of path direction across the 500 randomized model runs: * indicates that the proportion of coefficients with signs opposite to the mean coefficient was < 0.05; # indicates that this proportion was < 0.10; ns indicates this proportion was > 0.10. L_CV: local community coefficient of variation; R_CV: regional community coefficient of variation; R_Syn: regional community synchrony; L_Mean: local mean GPP; L_SD: local GPP standard deviation; L_Resistance: local community resistance; L_Recovery: local community recovery; L_FGR: local fast-growing phase growth rate; L_FGL: local fast-growing phase length; L_Prec: local precipitation; L_Temp: local temperature.

Table S11. R² values for meadow steppe

|  | Response variable | Mean R^2^ | Median R^2^ | R2 2.5% | R2 97.5% |
| --- | --- | --- | --- | --- | --- |
| 1.0° | R_CV | 1 | 1 | 1 | 1 |
|  | R_Syn | 0.48 | 0.47 | 0.03 | 0.95 |
|  | L_CV | 0.83 | 0.91 | 0.39 | 1 |
|  | L_Resistance | 0.41 | 0.38 | 0.05 | 0.94 |
|  | L_Recovery | 0.48 | 0.48 | 0.02 | 0.95 |
|  | L_Mean | 0.42 | 0.37 | 0.02 | 0.93 |
|  | L_SD | 0.79 | 0.84 | 0.31 | 0.99 |
|  | L_FGR | 0.40 | 0.42 | 0 | 0.87 |
|  | L_FGL | 0.38 | 0.36 | 0 | 0.87 |
| 0.5° | R_CV | 1 | 1 | 0.99 | 1 |
|  | R_Syn | 0.08 | 0.05 | 0 | 0.27 |
|  | L_CV | 0.65 | 0.66 | 0.43 | 0.80 |
|  | L_Resistance | 0.12 | 0.11 | 0.01 | 0.32 |
|  | L_Recovery | 0.21 | 0.20 | 0.03 | 0.47 |
|  | L_Mean | 0.71 | 0.72 | 0.50 | 0.85 |
|  | L_SD | 0.05 | 0.04 | 0 | 0.19 |
|  | L_FGR | 0.52 | 0.53 | 0.27 | 0.71 |
|  | L_FGL | 0.14 | 0.13 | 0.01 | 0.34 |
| 0.1° | R_CV | 0.99 | 0.99 | 0.99 | 0.99 |
|  | R_Syn | 0.04 | 0.04 | 0.01 | 0.07 |
|  | L_CV | 0.47 | 0.47 | 0.42 | 0.51 |
|  | L_Resistance | 0.08 | 0.08 | 0.05 | 0.11 |
|  | L_Recovery | 0.04 | 0.04 | 0.02 | 0.06 |
|  | L_Mean | 0.62 | 0.62 | 0.58 | 0.66 |
|  | L_SD | 0.11 | 0.11 | 0.08 | 0.15 |
|  | L_FGR | 0.38 | 0.38 | 0.33 | 0.43 |
|  | L_FGL | 0.13 | 0.13 | 0.09 | 0.17 |

Note: Values are R² values of component linear models in the piecewise SEM, calculated across the 500 randomized model runs. “Mean R^2^” represents the mean R², “Median R^2^” represents the median R², and “R^2^ 2.5%” and “R^2^ 97.5%” represent the 2.5th and 97.5th percentiles of the R² distribution, respectively. For each response variable, R² indicates the proportion of variance explained by its corresponding component model. R_CV: regional community coefficient of variation; R_Syn: regional community synchrony; L_CV: local community coefficient of variation; L_Mean: local mean GPP; L_SD: local GPP standard deviation; L_Resistance: local community resistance; L_Recovery: local community recovery; L_FGR: local fast-growing phase growth rate; L_FGL: local fast-growing phase length.

Table S12. R² values for typical steppe

|  | Response variable | Mean R^2^ | Median R^2^ | R2 2.5% | R2 97.5% |
| --- | --- | --- | --- | --- | --- |
| 1.0° | R_CV | 1 | 1 | 0.99 | 1 |
|  | R_Syn | 0.1 | 0.08 | 0 | 0.31 |
|  | L_CV | 0.79 | 0.79 | 0.64 | 0.90 |
|  | L_Resistance | 0.20 | 0.18 | 0.04 | 0.45 |
|  | L_Recovery | 0.07 | 0.05 | 0 | 0.23 |
|  | L_Mean | 0.87 | 0.88 | 0.74 | 0.96 |
|  | L_SD | 0.23 | 0.22 | 0.04 | 0.51 |
|  | L_FGR | 0.52 | 0.52 | 0.22 | 0.76 |
|  | L_FGL | 0.48 | 0.49 | 0.26 | 0.69 |
| 0.5° | R_CV | 0.99 | 0.99 | 0.99 | 1 |
|  | R_Syn | 0.04 | 0.03 | 0 | 0.12 |
|  | L_CV | 0.72 | 0.73 | 0.66 | 0.79 |
|  | L_Resistance | 0.30 | 0.30 | 0.18 | 0.45 |
|  | L_Recovery | 0.06 | 0.06 | 0.01 | 0.15 |
|  | L_Mean | 0.81 | 0.81 | 0.74 | 0.88 |
|  | L_SD | 0.22 | 0.21 | 0.11 | 0.34 |
|  | L_FGR | 0.49 | 0.50 | 0.35 | 0.63 |
|  | L_FGL | 0.23 | 0.23 | 0.12 | 0.35 |
| 0.1° | R_CV | 0.99 | 0.99 | 0.99 | 0.99 |
|  | R_Syn | 0.01 | 0.01 | 0 | 0.01 |
|  | L_CV | 0.65 | 0.65 | 0.63 | 0.66 |
|  | L_Resistance | 0.15 | 0.15 | 0.13 | 0.17 |
|  | L_Recovery | 0.01 | 0.01 | 0.01 | 0.02 |
|  | L_Mean | 0.82 | 0.82 | 0.80 | 0.83 |
|  | L_SD | 0.17 | 0.17 | 0.15 | 0.20 |
|  | L_FGR | 0.43 | 0.43 | 0.39 | 0.45 |
|  | L_FGL | 0.13 | 0.13 | 0.11 | 0.14 |

Note: Values are R² values of component linear models in the piecewise SEM, calculated across the 500 randomized model runs. “Mean R^2^” represents the mean R², “Median R^2^” represents the median R², and “R^2^ 2.5%” and “R^2^ 97.5%” represent the 2.5th and 97.5th percentiles of the R² distribution, respectively. For each response variable, R² indicates the proportion of variance explained by its corresponding component model. R_CV: regional community coefficient of variation; R_Syn: regional community synchrony; L_CV: local community coefficient of variation; L_Mean: local mean GPP; L_SD: local GPP standard deviation; L_Resistance: local community resistance; L_Recovery: local community recovery; L_FGR: local fast-growing phase growth rate; L_FGL: local fast-growing phase length.

Table S13. R² values for desert steppe

|  | Response variable | Mean R^2^ | Median R^2^ | R2 2.5% | R2 97.5% |
| --- | --- | --- | --- | --- | --- |
| 1.0° | R_CV | 1 | 1 | 1 | 1 |
|  | R_Syn | 0.48 | 0.49 | 0.03 | 0.93 |
|  | L_CV | 0.78 | 0.82 | 0.24 | 1 |
|  | L_Resistance | 0.46 | 0.44 | 0.05 | 0.92 |
|  | L_Recovery | 0.65 | 0.66 | 0.28 | 0.96 |
|  | L_Mean | 0.95 | 0.97 | 0.79 | 1 |
|  | L_SD | 0.67 | 0.69 | 0.34 | 0.93 |
|  | L_FGR | 0.59 | 0.61 | 0.12 | 0.96 |
|  | L_FGL | 0.46 | 0.47 | 0.01 | 0.88 |
| 0.5° | R_CV | 1 | 1 | 1 | 1 |
|  | R_Syn | 0.14 | 0.12 | 0.01 | 0.38 |
|  | L_CV | 0.68 | 0.69 | 0.51 | 0.83 |
|  | L_Resistance | 0.15 | 0.14 | 0.01 | 0.37 |
|  | L_Recovery | 0.20 | 0.18 | 0.02 | 0.46 |
|  | L_Mean | 0.91 | 0.91 | 0.79 | 0.97 |
|  | L_SD | 0.54 | 0.55 | 0.29 | 0.77 |
|  | L_FGR | 0.40 | 0.41 | 0.14 | 0.64 |
|  | L_FGL | 0.23 | 0.22 | 0.04 | 0.47 |
| 0.1° | R_CV | 1 | 1 | 1 | 1 |
|  | R_Syn | 0.01 | 0.01 | 0 | 0.03 |
|  | L_CV | 0.63 | 0.63 | 0.6 | 0.66 |
|  | L_Resistance | 0.09 | 0.09 | 0.06 | 0.12 |
|  | L_Recovery | 0.04 | 0.04 | 0.02 | 0.06 |
|  | L_Mean | 0.88 | 0.88 | 0.86 | 0.90 |
|  | L_SD | 0.34 | 0.34 | 0.29 | 0.39 |
|  | L_FGR | 0.15 | 0.15 | 0.11 | 0.2 |
|  | L_FGL | 0.16 | 0.15 | 0.12 | 0.20 |

Note: Values are R² values of component linear models in the piecewise SEM, calculated across the 500 randomized model runs. “Mean R^2^” represents the mean R², “Median R^2^” represents the median R², and “R^2^ 2.5%” and “R^2^ 97.5%” represent the 2.5th and 97.5th percentiles of the R² distribution, respectively. For each response variable, R² indicates the proportion of variance explained by its corresponding component model. R_CV: regional community coefficient of variation; R_Syn: regional community synchrony; L_CV: local community coefficient of variation; L_Mean: local mean GPP; L_SD: local GPP standard deviation; L_Resistance: local community resistance; L_Recovery: local community recovery; L_FGR: local fast-growing phase growth rate; L_FGL: local fast-growing phase length.

Table S14. Statistical power analysis based on the number of pixel-year drought observations

| Steppe type | Grain size | Number of pixel-year drought observations | Power for r = 0.5 | Power for r = 0.6 |
| --- | --- | --- | --- | --- |
| Meadow | 1.0° | 16 | 0.53 | 0.73 |
|  | 0.5° | 62 | 0.99 | 1.00 |
|  | 0.1° | 1235 | 1.00 | 1.00 |
| Typical | 1.0° | 43 | 0.94 | 0.99 |
|  | 0.5° | 165 | 1.00 | 1.00 |
|  | 0.1° | 3931 | 1.00 | 1.00 |
| Desert | 1.0° | 16 | 0.53 | 0.73 |
|  | 0.5° | 62 | 0.99 | 0.99 |
|  | 0.1° | 1406 | 1.00 | 1.00 |

Note: Statistical power was calculated for detecting Pearson correlations of r = 0.5 and r = 0.6 using a two-sided test with α = 0.05. The number of pixel-year drought observations corresponds to the drought-observation sample size used for each steppe type and grain size. Power values below 0.80 indicate limited statistical power.

## **Supplementary Figures**

**Figure S1** Monthly averages of environmental variables for Inner Mongolian grasslands and different steppe types from 2000 to 2018

**Figure S2** Regression relationships between regional and local community CV across different steppe types and spatial resolutions in Inner Mongolia. Colored lines represent the regression fitted across all regional communities pooled together. Gray lines represent regression fits for each of the 500 regional communities constructed from non-repetitive random combinations of local communities. n denotes the number of local communities used to construct regional units. $\bar{R}^{2}$ and $\bar{p}$ represent the mean coefficient of determination and *p*-value across the 500 replicates, respectively. Solid lines indicate statistically significant relationships (*p* < 0.05); dashed lines denote nonsignificant relationships (*p* > 0.05). CV denotes the coefficient of variation, used here as a metric of temporal stability.

**Figure S3** Regression relationships between regional community CV and synchrony across different steppe types and spatial resolutions in Inner Mongolia. Colored lines represent the regression fitted across all regional communities pooled together. Gray lines represent regression fits for each of the 500 regional communities constructed from non-repetitive random combinations of local communities. n denotes the number of local communities used to construct regional units. $\bar{R}^{2}$ and $\bar{p}$ represent the mean coefficient of determination and *p*-value across the 500 replicates, respectively. Solid lines indicate statistically significant relationships (*p* < 0.05); dashed lines denote nonsignificant relationships (*p* > 0.05). CV denotes the coefficient of variation, used here as a metric of temporal stability.

**Figure S4** Regression relationships between local community CV and GPP across different steppe types and spatial resolutions in Inner Mongolia. Colored lines represent the regression fitted across all regional communities pooled together. Gray lines represent regression fits for each of the 500 regional communities constructed from non-repetitive random combinations of local communities. n denotes the number of local communities used to construct regional units. $\bar{R}^{2}$ and $\bar{p}$ represent the mean coefficient of determination and *p*-value across the 500 replicates, respectively. Solid lines indicate statistically significant relationships (*p* < 0.05); dashed lines denote nonsignificant relationships (*p* > 0.05). CV denotes the coefficient of variation, used here as a metric of temporal stability. GPP, gross primary productivity.

**Figure S5** Regression relationships between local community CV and GPP SD across different steppe types and spatial resolutions in Inner Mongolia. Colored lines represent the regression fitted across all regional communities pooled together. Gray lines represent regression fits for each of the 500 regional communities constructed from non-repetitive random combinations of local communities. n denotes the number of local communities used to construct regional units. $\bar{R}^{2}$ and $\bar{p}$ represent the mean coefficient of determination and *p*-value across the 500 replicates, respectively. Solid lines indicate statistically significant relationships (*p* < 0.05); dashed lines denote nonsignificant relationships (*p* > 0.05). CV denotes the coefficient of variation, used here as a metric of temporal stability. GPP, gross primary productivity. SD, standard deviation.

**Figure S6** Regression relationships between local community CV and resistance across different steppe types and spatial resolutions in Inner Mongolia. Colored lines represent the regression fitted across all regional communities pooled together. Gray lines represent regression fits for each of the 500 regional communities constructed from non-repetitive random combinations of local communities. n denotes the number of local communities used to construct regional units. $\bar{R}^{2}$ and $\bar{p}$ represent the mean coefficient of determination and *p*-value across the 500 replicates, respectively. Solid lines indicate statistically significant relationships (*p* < 0.05); dashed lines denote nonsignificant relationships (*p* > 0.05). CV denotes the coefficient of variation, used here as a metric of temporal stability. GPP, gross primary productivity.

**Figure S7** Regression relationships between local community CV and recovery across different steppe types and spatial resolutions in Inner Mongolia. Colored lines represent the regression fitted across all regional communities pooled together. Gray lines represent regression fits for each of the 500 regional communities constructed from non-repetitive random combinations of local communities. n denotes the number of local communities used to construct regional units. $\bar{R}^{2}$ and $\bar{p}$ represent the mean coefficient of determination and *p*-value across the 500 replicates, respectively. Solid lines indicate statistically significant relationships (*p* < 0.05); dashed lines denote nonsignificant relationships (*p* > 0.05). CV denotes the coefficient of variation, used here as a metric of temporal stability. GPP, gross primary productivity.

**Figure S8** Regression relationships between local community resistance and GPP across different steppe types and spatial resolutions in Inner Mongolia. Colored lines represent the regression fitted across all regional communities pooled together. Gray lines represent regression fits for each of the 500 regional communities constructed from non-repetitive random combinations of local communities. n denotes the number of local communities used to construct regional units. $\bar{R}^{2}$ and $\bar{p}$ represent the mean coefficient of determination and *p*-value across the 500 replicates, respectively. Solid lines indicate statistically significant relationships (*p* < 0.05); dashed lines denote nonsignificant relationships (*p* > 0.05). CV denotes the coefficient of variation, used here as a metric of temporal stability. GPP, gross primary productivity.

**Figure S9** Regression relationships between local community resistance and GPP SD across different steppe types and spatial resolutions in Inner Mongolia. Colored lines represent the regression fitted across all regional communities pooled together. Gray lines represent regression fits for each of the 500 regional communities constructed from non-repetitive random combinations of local communities. n denotes the number of local communities used to construct regional units. $\bar{R}^{2}$ and $\bar{p}$ represent the mean coefficient of determination and *p*-value across the 500 replicates, respectively. Solid lines indicate statistically significant relationships (*p* < 0.05); dashed lines denote nonsignificant relationships (*p* > 0.05). CV denotes the coefficient of variation, used here as a metric of temporal stability. GPP, gross primary productivity. SD, standard deviation.

**Figure S10** Regression relationships between local community recovery and GPP across different steppe types and spatial resolutions in Inner Mongolia. Colored lines represent the regression fitted across all regional communities pooled together. Gray lines represent regression fits for each of the 500 regional communities constructed from non-repetitive random combinations of local communities. n denotes the number of local communities used to construct regional units. $\bar{R}^{2}$ and $\bar{p}$ represent the mean coefficient of determination and *p*-value across the 500 replicates, respectively. Solid lines indicate statistically significant relationships (*p* < 0.05); dashed lines denote nonsignificant relationships (*p* > 0.05). CV denotes the coefficient of variation, used here as a metric of temporal stability. GPP, gross primary productivity.

**Figure S11** Regression relationships between local community recovery and GPP SD across different steppe types and spatial resolutions in Inner Mongolia. Colored lines represent the regression fitted across all regional communities pooled together. Gray lines represent regression fits for each of the 500 regional communities constructed from non-repetitive random combinations of local communities. n denotes the number of local communities used to construct regional units. $\bar{R}^{2}$ and $\bar{p}$ represent the mean coefficient of determination and *p*-value across the 500 replicates, respectively. Solid lines indicate statistically significant relationships (*p* < 0.05); dashed lines denote nonsignificant relationships (*p* > 0.05). CV denotes the coefficient of variation, used here as a metric of temporal stability. GPP, gross primary productivity. SD, standard deviation.

**Figure S12** Regression relationships between local community GPP and FGR across different steppe types and spatial resolutions in Inner Mongolia. Colored lines represent the regression fitted across all regional communities pooled together. Gray lines represent regression fits for each of the 500 regional communities constructed from non-repetitive random combinations of local communities. n denotes the number of local communities used to construct regional units. $\bar{R}^{2}$ and $\bar{p}$ represent the mean coefficient of determination and *p*-value across the 500 replicates, respectively. Solid lines indicate statistically significant relationships (*p* < 0.05); dashed lines denote nonsignificant relationships (*p* > 0.05). CV denotes the coefficient of variation, used here as a metric of temporal stability. GPP, gross primary productivity. FGR, growth rate of the fast-growing phase.

**Figure S13** Regression relationships between local community GPP and FGL across different steppe types and spatial resolutions in Inner Mongolia. Colored lines represent the regression fitted across all regional communities pooled together. Gray lines represent regression fits for each of the 500 regional communities constructed from non-repetitive random combinations of local communities. n denotes the number of local communities used to construct regional units. $\bar{R}^{2}$ and $\bar{p}$ represent the mean coefficient of determination and *p*-value across the 500 replicates, respectively. Solid lines indicate statistically significant relationships (*p* < 0.05); dashed lines denote nonsignificant relationships (*p* > 0.05). CV denotes the coefficient of variation, used here as a metric of temporal stability. GPP, gross primary productivity. FGL, duration of the fast-growing phase.

**Figure S14** Regression relationships between local community GPP SD and FGR across different steppe types and spatial resolutions in Inner Mongolia. Colored lines represent the regression fitted across all regional communities pooled together. Gray lines represent regression fits for each of the 500 regional communities constructed from non-repetitive random combinations of local communities. n denotes the number of local communities used to construct regional units. $\bar{R}^{2}$ and $\bar{p}$ represent the mean coefficient of determination and *p*-value across the 500 replicates, respectively. Solid lines indicate statistically significant relationships (*p* < 0.05); dashed lines denote nonsignificant relationships (*p* > 0.05). CV denotes the coefficient of variation, used here as a metric of temporal stability. GPP, gross primary productivity. SD, standard deviation. FGR, growth rate of the fast-growing phase.

**Figure S15** Regression relationships between local community GPP SD and FGR across different steppe types and spatial resolutions in Inner Mongolia. Colored lines represent the regression fitted across all regional communities pooled together. Gray lines represent regression fits for each of the 500 regional communities constructed from non-repetitive random combinations of local communities. n denotes the number of local communities used to construct regional units. $\bar{R}^{2}$ and $\bar{p}$ represent the mean coefficient of determination and *p*-value across the 500 replicates, respectively. Solid lines indicate statistically significant relationships (*p* < 0.05); dashed lines denote nonsignificant relationships (*p* > 0.05). CV denotes the coefficient of variation, used here as a metric of temporal stability. GPP, gross primary productivity. SD, standard deviation. FGL, duration of the fast-growing phase.

**Figure S16** Regression relationships between local community FGR and precipitation across different steppe types and spatial resolutions in Inner Mongolia. Colored lines represent the regression fitted across all regional communities pooled together. Gray lines represent regression fits for each of the 500 regional communities constructed from non-repetitive random combinations of local communities. n denotes the number of local communities used to construct regional units. $\bar{R}^{2}$ and $\bar{p}$ represent the mean coefficient of determination and *p*-value across the 500 replicates, respectively. Solid lines indicate statistically significant relationships (*p* < 0.05); dashed lines denote nonsignificant relationships (*p* > 0.05). CV denotes the coefficient of variation, used here as a metric of temporal stability. GPP, gross primary productivity. FGR, growth rate of the fast-growing phase.

**Figure S17** Regression relationships between local community FGR and temperature across different steppe types and spatial resolutions in Inner Mongolia. Colored lines represent the regression fitted across all regional communities pooled together. Gray lines represent regression fits for each of the 500 regional communities constructed from non-repetitive random combinations of local communities. n denotes the number of local communities used to construct regional units. $\bar{R}^{2}$ and $\bar{p}$ represent the mean coefficient of determination and *p*-value across the 500 replicates, respectively. Solid lines indicate statistically significant relationships (*p* < 0.05); dashed lines denote nonsignificant relationships (*p* > 0.05). CV denotes the coefficient of variation, used here as a metric of temporal stability. GPP, gross primary productivity. FGR, growth rate of the fast-growing phase.

**Figure S18** Regression relationships between local community FGR and solar radiation across different steppe types and spatial resolutions in Inner Mongolia. Colored lines represent the regression fitted across all regional communities pooled together. Gray lines represent regression fits for each of the 500 regional communities constructed from non-repetitive random combinations of local communities. n denotes the number of local communities used to construct regional units. $\bar{R}^{2}$ and $\bar{p}$ represent the mean coefficient of determination and *p*-value across the 500 replicates, respectively. Solid lines indicate statistically significant relationships (*p* < 0.05); dashed lines denote nonsignificant relationships (*p* > 0.05). CV denotes the coefficient of variation, used here as a metric of temporal stability. GPP, gross primary productivity. FGR, growth rate of the fast-growing phase.

**Figure S19** Regression relationships between local community FGR and soil moisture across different steppe types and spatial resolutions in Inner Mongolia. Colored lines represent the regression fitted across all regional communities pooled together. Gray lines represent regression fits for each of the 500 regional communities constructed from non-repetitive random combinations of local communities. n denotes the number of local communities used to construct regional units. $\bar{R}^{2}$ and $\bar{p}$ represent the mean coefficient of determination and *p*-value across the 500 replicates, respectively. Solid lines indicate statistically significant relationships (*p* < 0.05); dashed lines denote nonsignificant relationships (*p* > 0.05). CV denotes the coefficient of variation, used here as a metric of temporal stability. GPP, gross primary productivity. FGR, growth rate of the fast-growing phase.

**Figure S20** Regression relationships between local community FGL and precipitation across different steppe types and spatial resolutions in Inner Mongolia. Colored lines represent the regression fitted across all regional communities pooled together. Gray lines represent regression fits for each of the 500 regional communities constructed from non-repetitive random combinations of local communities. n denotes the number of local communities used to construct regional units. $\bar{R}^{2}$ and $\bar{p}$ represent the mean coefficient of determination and *p*-value across the 500 replicates, respectively. Solid lines indicate statistically significant relationships (*p* < 0.05); dashed lines denote nonsignificant relationships (*p* > 0.05). CV denotes the coefficient of variation, used here as a metric of temporal stability. GPP, gross primary productivity. FGL, duration of the fast-growing phase.

**Figure S21** Regression relationships between local community FGL and temperature across different steppe types and spatial resolutions in Inner Mongolia. Colored lines represent the regression fitted across all regional communities pooled together. Gray lines represent regression fits for each of the 500 regional communities constructed from non-repetitive random combinations of local communities. n denotes the number of local communities used to construct regional units. $\bar{R}^{2}$ and $\bar{p}$ represent the mean coefficient of determination and *p*-value across the 500 replicates, respectively. Solid lines indicate statistically significant relationships (*p* < 0.05); dashed lines denote nonsignificant relationships (*p* > 0.05). CV denotes the coefficient of variation, used here as a metric of temporal stability. GPP, gross primary productivity. FGL, duration of the fast-growing phase.

**Figure S22** Regression relationships between local community FGL and solar radiation across different steppe types and spatial resolutions in Inner Mongolia. Colored lines represent the regression fitted across all regional communities pooled together. Gray lines represent regression fits for each of the 500 regional communities constructed from non-repetitive random combinations of local communities. n denotes the number of local communities used to construct regional units. $\bar{R}^{2}$ and $\bar{p}$ represent the mean coefficient of determination and *p*-value across the 500 replicates, respectively. Solid lines indicate statistically significant relationships (*p* < 0.05); dashed lines denote nonsignificant relationships (*p* > 0.05). CV denotes the coefficient of variation, used here as a metric of temporal stability. GPP, gross primary productivity. FGL, duration of the fast-growing phase.

**Figure S23** Regression relationships between local community FGL and soil moisture across different steppe types and spatial resolutions in Inner Mongolia. Colored lines represent the regression fitted across all regional communities pooled together. Gray lines represent regression fits for each of the 500 regional communities constructed from non-repetitive random combinations of local communities. n denotes the number of local communities used to construct regional units. $\bar{R}^{2}$ and $\bar{p}$ represent the mean coefficient of determination and *p*-value across the 500 replicates, respectively. Solid lines indicate statistically significant relationships (*p* < 0.05); dashed lines denote nonsignificant relationships (*p* > 0.05). CV denotes the coefficient of variation, used here as a metric of temporal stability. GPP, gross primary productivity. FGL, duration of the fast-growing phase.
